# SARS-CoV-2 spike fusion peptide *trans* interaction with phosphatidylserine lipid triggers membrane fusion for viral entry

**DOI:** 10.1128/mbio.01077-24

**Published:** 2024-08-08

**Authors:** Puspangana Singh, Purba Pahari, Srija Mukherjee, Sharmistha Karmakar, Markus Hoffmann, Taraknath Mandal, Dibyendu Kumar Das

**Affiliations:** 1 Department of Biological Sciences and Bioengineering, Indian Institute of Technology Kanpur, Kanpur, Uttar Pradesh, India; 2 Department of Chemical and Biomolecular Engineering, University of Maryland, College Park, Maryland, USA; 3 Infection Biology Unit, German Primate Center—Leibniz Institute for Primate Research, Göttingen, Germany; 4 Faculty of Biology and Psychology, Georg August University, Göttingen, Germany; 5 Department of Physics, Indian Institute of Technology Kanpur, Kanpur, Uttar Pradesh, India; 6 Center for Engineering in Medicine, Indian Institute of Technology Kanpur, Kanpur, Uttar Pradesh, India; Columbia University Medical College, New York, USA

**Keywords:** SARS-CoV-2, virus entry, conformational dynamics, membrane fusion, smFRET, lipid

## Abstract

Severe acute respiratory syndrome coronavirus 2 (SARS-CoV-2) spike is the fusion machine for host cell entry. Still, the mechanism by which spike protein interacts with the target lipid membrane to facilitate membrane fusion during entry is not fully understood. Here, using steady-state membrane fusion and single-molecule fluorescence resonance energy transfer imaging of spike trimers on the surface of SARS-CoV-2 pseudovirion, we directly show that spike protein interacts with phosphatidylserine (PS) lipid in the target membrane for mediating fusion. We observed that the fusion peptide of the spike S2 domain interacts with the PS lipid of the target membrane. Low pH and Ca^2+^ trigger the spike conformational change and bring fusion peptide in close proximity to the PS lipid of the membrane. The binding of the spike with PS lipid of its viral membrane (*cis* interaction) impedes the fusion activation. PS on the target membrane promotes spike binding via *trans* interaction, prevents the *cis* interaction, and accelerates fusion. Sequestering or absence of PS lipid abrogates the spike-mediated fusion process and restricts SARS-CoV-2 infectivity. We found that PS-dependent interaction for fusion is conserved across all the SARS-CoV-2 spike variants of concern (D614G, Alpha, Beta, Delta, and Omicron). Our study suggests that PS lipid is indispensable for SARS-CoV-2 spike-mediated virus and target membrane fusion for entry, and restricting PS interaction with spike inhibits the SARS-CoV-2 spike-mediated entry. Therefore, PS is an important cofactor and acts as a molecular beacon in the target membrane for SARS-CoV-2 entry.

**IMPORTANCE:**

The role of lipids in the host cell target membrane for severe acute respiratory syndrome coronavirus 2 (SARS-CoV-2) entry is not clear. We do not know whether SARS-CoV-2 spike protein has any specificity in terms of lipid for membrane fusion reaction. Here, using *in vitro* reconstitution of membrane fusion assay and single-molecule fluorescence resonance energy transfer imaging of SARS-CoV-2 spike trimers on the surface of the virion, we have demonstrated that phosphatidylserine (PS) lipid plays a key role in SARS-CoV-2 spike-mediated membrane fusion reaction for entry. Membrane-externalized PS lipid strongly promotes spike-mediated membrane fusion and COVID-19 infection. Blocking externalized PS lipid with PS-binding protein or in the absence of PS, SARS-CoV-2 spike-mediated fusion is strongly inhibited. Therefore, PS is an important target for restricting viral entry and intervening spike, and PS interaction presents new targets for COVID-19 interventions.

## INTRODUCTION

Recent and ongoing outbreaks of severe acute respiratory syndrome coronavirus 2 (SARS-CoV-2) caused COVID-19 infection worldwide, which have resulted in unprecedented loss of life (1). The persistent threat of COVID-19 infection underscores the importance of understanding the mechanism by which SARS-CoV-2 enters cells. Understanding the viral entry process will facilitate the establishment of new strategies for therapeutic and preventative measures. For initiation of infection, SARS-CoV-2 delivers its RNA into the cell by fusing the viral membrane with the cell membrane (2, 3). The fusion process is mediated by the trimeric spike (S) glycoprotein. The S protein is processed by host cell furin protease into the receptor-binding subunit S1 and the fusion subunit S2 (4, 5). Engagement of host cell receptors ACE2, NRP1 with the S1 domain of spike, initiates docking, and endocytosis of the virus followed by a second proteolytic cleavage within S2 domain (S2′ site) for fusion between viral membrane and endosomal membrane (6–10). The TMPRSS2-mediated cleavage activates SARS-CoV-2 S protein for fusion at neutral pH (6, 7, 10, 11*).* The cleaved spike undergoes large conformational changes, resulting in the dissociation of S1 and structural refolding of the S2 into a post-fusion structure to trigger the fusion of the virus and host cell membranes (10–12). Receptor and spike interaction is essential for SARS-CoV-2 entry, but how this interaction activates the S2 domain for fusion is unknown. What is the role of lipids in the target membrane for S2-mediated fusion is unclear.

Recent studies have suggested that the SARS-CoV-2 spike is a calcium sensor and spike-mediated fusion and infection also depend on intracellular calcium signaling (13, 14). Specifically, the Ca^2+^ signaling is triggered by the engagement of viral spike protein with the cellular receptors. Intracellular Ca^2+^ rise also triggers a redistribution of phosphatidylserine (PS) lipid from the inner leaflet to the outer leaflet of the plasma membrane via Ca^2+^-activated chloride channels or scramblases, TMEM16F (14–16). Now, PS lipid exposure is known for cell fusion events like macrophage fusing into inflammatory giant cells (17) or to form bone reabsorbing osteoclasts (18), myoblast fusing to myotubes (19), and sperm cell fusion for egg fertilization (20). However, the role of PS lipid in SARS-CoV-2 spike-mediated membrane fusion is not known.

The role of lipids in the target membrane for SARS-CoV-2 spike-mediated fusion reaction for entry is not clear. We do not know whether spike protein has any specificity in terms of lipid for fusion reaction. Cell membrane lipids have been known to interact with other enveloped viral glycoproteins. Entry of other enveloped viruses such as HIV, dengue, Ebola, and VSV depends on the PS lipid exposure in the outer surface of the host cell membrane (21–25). A recent study has shown that blocking the calcium ion channel of TMEM16F, which mediates PS externalization, results in reduction in SARS-CoV-2 spike-mediated syncytia formation (14). It has also been reported that PS receptor proteins on the host cell (TIM and TAM) enhance SARS-CoV-2 entry (26). As the interaction of PS lipid with SARS-CoV-2 spike has not been directly demonstrated, the role of PS in SARS-CoV-2 entry remains enigmatic. Hence, it is important to understand the mechanisms through which PS lipid interacts with the SARS-CoV-2 spike trimers for promoting fusion. Delineating this interaction would help in designing therapeutic strategies for intervening in COVID-19 infections.

Here, using *in vitro* reconstitution of membrane fusion assay and single-molecule FRET imaging of SARS-CoV-2 spike trimers on the surface of virions, we have demonstrated that phosphatidylserine lipid is indispensable for SARS-CoV-2 spike-mediated membrane fusion reaction for entry. Membrane-externalized PS lipid strongly promotes spike-mediated membrane fusion and COVID-19 infection. Blocking externalized PS lipid with PS-binding protein or in the absence of PS, SARS-CoV-2 spike-mediated fusion is strongly inhibited. Exogenous PS added to the plasma membrane promoted membrane fusion and viral entry. To probe the role of PS in membrane fusion, we designed a fluorescence dequenching-based fusion assay between SARS-CoV-2 pseudovirion for Omicron, Delta, Alpha, Beta, and D614G strains and proteoliposome coated with the receptors, ACE2, NRP1, and TMPRSS2 protease. Across all the spike variants, we find that membrane fusion requires PS lipid in the target liposome. Ca^2+^ concentration (500 μM) and low pH facilitate the spike and PS interaction for fusion. Furthermore, to probe the direct interaction between the spike and PS, we designed a novel single-molecule fluorescence resonance energy transfer (smFRET) imaging assay, where a single spike trimer of the SARS-CoV-2 pseudovirion of Delta or D614G spike variants was genetically labeled with an acceptor fluorophore, using amber stop codon suppression techniques and the PS lipid was labeled with the donor fluorophore. Our smFRET analysis suggests that the fusion peptide of spike S2 domain of intact spike protein directly interacts with PS lipid to promote fusion. This spike protein and PS lipid interaction is calcium dependent, suggesting that Ca^2+^ coordinates the PS and fusion peptide interaction for fusion. This spike and PS interactions can take place either in the same side (*cis*) or in the *trans* pathway. In *cis* pathways, the viral spike interacts with its PS lipid in the virion membrane and inactivates the membrane fusion whereas the *trans* interaction between spike and PS led to the activation of membrane fusion and spike prefusion conformation converts into an irreversible post-fusion state. We found that the SARS-CoV-2 spike has evolved to be a reversible fusion machine so that it can rescue the spike protein by reversible conformational change after *cis* interaction with PS lipid. Therefore, PS is an important target for restricting the viral entry and intervening spike and PS interaction presents new targets for COVID-19 interventions.

## RESULTS

### Reconstitution of SARS-CoV-2 spike-mediated viral and liposome membrane fusion requires PS lipid

**Fig 1:**
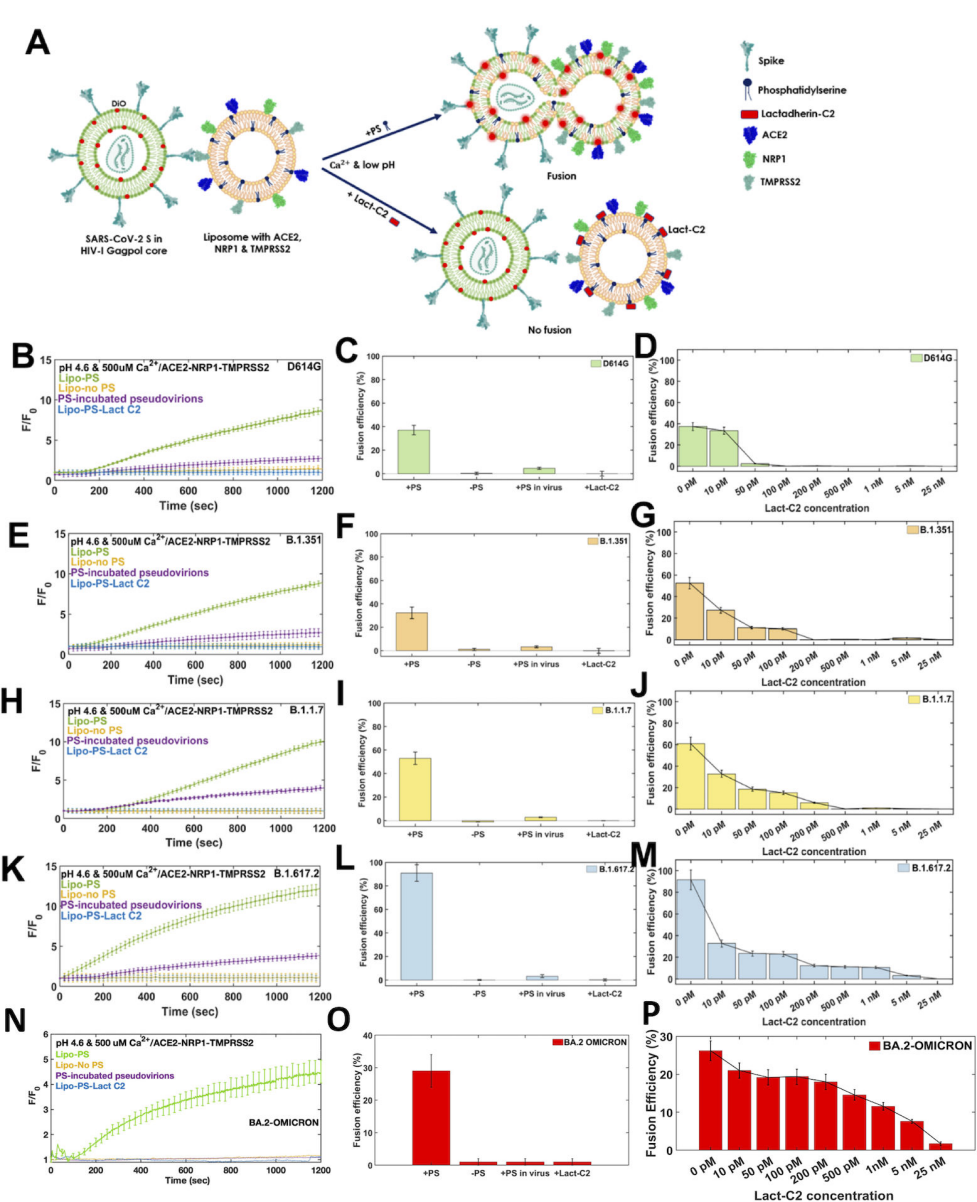
SARS-CoV-2 spike-mediated virus-proteoliposome membrane fusion is PS lipid dependent. (**A**) Illustration of the steady-state fusion assay between SARS-CoV-2 spike pseudovirion membrane and liposome membrane based on fluorescence dequenching. SARS-CoV-2 pseudovirions were labeled with the lipophilic dye DiO. Liposomes were coated with recombinant ACE2, NRP1, and TMPRSS2 proteins, and the fusion reaction was followed for different cases at pH 4.6 and 500 μM Ca^2+^, for the (**B, C, D**) D614G spike pseudovirions, (**E, F, G**) B.1.351 spike pseudovirions, (**H, I, J**) B.1.1.7 spike pseudovirions, (**K, L, M**) B.1.617.2 spike, and (**N, O, P**) B.1.1.529 spike pseudovirions. Under optimal conditions, when liposomes contain PS lipid and were coated with the receptors, robust DiO dequenching was observed as a result of lipid mixing proceeding for full fusion for all the spike variants (green lines in the F/F_0_ plots) (**B, E, H, K, N**). In the absence of PS in the liposome, no fluorescence dequenching or lipid mixing was observed (yellow lines in the F/F_0_ plots). When liposomes were coated with recombinant Lact-C2 protein, no DiO dequenching was observed (blue lines in the F/F_0_ plots). In a separate set of experiment, when virions were incubated with 1 μM exogenous PS, and the fusion efficiency was considerably reduced for all the strains (purple lines in the F/F_0_ plots). (**C, F, I, L, O**) The fusion efficiency has been plotted for indicated conditions for spike variants (D614G/Alpha/Beta/Delta/Omicron variants). The error bars represent the data from a set of triplicates for each experiment (*N* = 3). For calculating fusion efficiency, once the reaction was followed for 20 minutes, 0.1% Triton-X was added to the fusion reaction to get the maximum fluorescence readout of the DiO from the virions due to complete fusion (Materials and Methods)**.** (**D, G, J, M, P**) The virus-liposome fusion efficiency in the presence of varying Lact-C2 concentration (10 pM to 25 nM) for D614G, B.1.351, B.1.1.7, B.1.617.2, and B.1.1.529 pseudovirions, respectively. The pseudovirions were labeled with DiO, and liposomes were coated with varying concentrations of recombinant Lact-C2 protein, as per indicated. The fusion reaction was then carried out in the presence of low pH and Ca^2+^as described in Materials and Methods. The fusion efficiency in the presence of varying concentrations of Lact-C2 has been plotted for the spike strains (D614G/Alpha/Beta/Delta/Omicron variants).

To obtain direct evidence for PS-dependent fusion, we performed *in vitro* fusion assay between the pseudovirions and liposomes coated with receptors ACE2, NRP1, and TMPRSS2 protease. We produced SARS-CoV-2 pseudotyped virions with the different spike variants of concern (D614G/B.1.351/B.1.1.7/B.1.617.2/B.1.1.529), with the retroviral core of HIV-1 (human immunodeficiency virus) (Fig. 1A). The viral membrane was labeled with a lipophilic dye, DiO. The labeled virions were then introduced to liposomes, which had PS lipid along with other lipids such as DOPC, POPC, Ni-NTA DGS lipid, and cholesterol. The liposomes were coated with ACE2, NRP1, and TMPRSS2 (Fig. 1A). The fusion reaction was carried out in the presence of 500 μM Ca^2+^ and pH 4.6, as it has been determined to be the triggering factor for SARS-CoV-2 fusion (13). Fusion was observed in the form of DiO fluorescence dequenching resulting from lipid mixing between the viral and the liposome membranes (Fig. 1B). For D614G spike variants, a clear dequenching signal was observed (indicated by the green curve in Fig. 1B) in the presence of PS liposomes coated with the known receptors. A similar fusion assay was performed for the D614G spike virion with liposome having no PS lipid, and remarkably, no dequenching signal was obtained (indicated by the yellow curve in Fig. 1B). Furthermore, when pseudovirions were pre-incubated with exogenous PS in the fusion assay, fusion efficiency was reduced to a considerable extent (purple curve in Fig. 1B; Fig. S1). For D614G spike, the efficiency was reduced from around 40% (in the presence of PS liposomes coated with the receptors) to around 3% when the pseudovirions were incubated with PS (Fig. 1C). Finally, we introduced recombinant Lact-C2 protein to the liposomes having PS, the C2 domain of Lactadherin binds to phosphatidylserine (PS) present on the liposomal membrane (27, 28), (Fig. 1A), and no dequenching due to fusion is observed (blue curve) (Fig. 1B–D; Fig. S1). Negative control experiments were performed between spike less pseudovirions and preoteoliposomes and between spike pseudovirions and liposomes without receptors, and no fluorescence dequenching was observed (Fig. S2). Positive control experiments with VSV-G pseudovirions showed appreciable fluorescence dequenching signal and PS-dependent membrane fusion activity (Fig. S2). These control data further strengthen our observation that SARS-CoV-2 spike-mediated fusion is PS lipid dependent.

This indicated that SARS-CoV-2 spike-mediated lipid mixing for fusion is dependent on PS lipid of the target liposome. Overall, all four experiments signify the role of PS in spike-mediated membrane fusion (Fig. 1C). Furthermore, we have titrated the fusion efficiency for D614G spike pseudovirion at varying concentrations of LactC2 protein (Fig. 1D). We found that, with increased in LactC2 concentration, the D614G spike-mediated fusion efficiency decreases (Fig. 1D). Similar sets of fusion experiments were carried out for B.1.351, B.1.1.7, B.1.617.2, and B.1.1.529.2 spike variants to understand the role of PS lipid in the spike-mediated fusion (Fig. 1E–P; Fig. S1). For all these spike variants, robust fusion is observed with liposomes having PS lipids. Lacking PS lipid in liposomes completely inhibits fusion for all the spike variants of concern (D614G to Omicron). Sequestering PS with LactC2 protein abrogates the spike mediated-membrane fusion in a concentration-dependent manner (Fig. 1G, J, M, and P; Fig. S3). Also, preincubating the PS with spike virion lowers the fusion efficiency, suggesting that PS interaction with spike may promote the pre- to post-fusion conformation change. To confirm that, Lact-C2 inhibits the spike fusion by interacting with PS only, we produced mutant Lact-C2, which is unable to bind the PS. The hydrophobic domain of Lact-C2 (26–28 residue WGL) was mutated to (AAA) to diminish the PS binding (Fig. S5) (29). The mutant Lact-C2 could not inhibit the spike fusion efficiency, observed for BA.2 and BA.3 omicron spike variants (Fig. S5). As control experiments, we have also performed the SARS-CoV-2 spike and liposome fusion measurements in the presence of the PE-binding protein, PEBP1. For both the pseudovirions (B.1.617.2 spike, and D614G spike), we did not observe any fusion inhibitory effect of PEBP protein in the spike fusion reaction (Fig. S6). For B.1.617.2 spike pseudovirions, approximately 90% fusogenicity was observed in the presence of PEBP1 protein, and for the D614G spike pseudovirions, approximately 35% fusogenicity was observed in the presence of PEBP protein (Fig. S6A). These control experiments demonstrate that PE-binding protein does not interfere with the fusion reaction.

### Sequestering PS with LactC2 restricts SARS-CoV-2 fusogenicity

To delineate the dependence of SARS-CoV-2 fusion on the presence of PS on the cell membrane, we performed a fusogenicity assay with pseudovirions using beta-lactamase enzyme-based assay (Fig. S7) (Materials and Methods) (30). Vero-TMPRSS2 cells were used as target cells for assessing the degree of fusogenicity of the pseudovirions under different conditions. B.1.617.2 pseudovirions showed the highest fusogenicity, followed by B.1.1.7, B.1.351, and D614G (Fig. S7). The expression of LactC2 in the target cells reduced the fusogenicity of the virions, consistent with our *in vitro* membrane fusion data (Fig. S2). In addition, incubation of cells with recombinant Lact-C2 again resulted in reduced fusogenicity. Prior incubation of the virions with PS lipid inhibited viral fusion to the target cells (Fig. S7). This indicated that PS could bind to the viral spike and prevent its interaction with the host cell membrane. Incubation of target cells with recombinant LactC2 inhibits the viral fusogenicity, but the addition of external PS rescued the suppressed viral fusogenicity (Fig. S7). Interestingly, incubation of the target cells with external PS resulted in increased SARS-CoV-2 fusogenicity.

### Lipid flotation assay and anisotropy assay for spike and PS interaction

Next, we have performed two separate assays to probe the spike-PS interaction. First, we have designed the lipid flotation assay to assess the binding of the spike protein to PS lipid (Fig. S8A). Two sets of liposomes were prepared, one having NBD-PS lipid and the other having rhodamine-PE lipid. Liposomes were incubated with spike pseudovirion for 45 minutes. The mixture was then subjected to purification using a 6%–30% Opti-Prep gradient. After density gradient centrifugation, the colored fraction having the liposome was collected and subjected to western blot. The primary antibody against the spike was used to detect the presence of the spike in the liposome sample. The western blot data (Fig. S8B) show that both the B.1.617.2 and D614G spike have bound only to liposomes having phosphatidylserine, and no spike was detected with the PE liposomes. Similarly, we have performed the lipid flotation assay with recombinant B.1.617.2 spike protein and Omicron spike protein and found that both the omicron and B.1.617.2 spike were detected in the presence of PS liposome only (Fig. S8B). No spike protein was detected in the absence of PS lipids in the liposomes. This suggests the specific binding of spike and PS lipids.

Second, to determine the binding of PS lipid with the SARS-CoV-2 spike protein, we developed a fluorescence-based anisotropy assay to track the mobility of the fusion peptide (Fig. S8D and E). Anisotropy can determine the free state to bound state conformation change of spike during interaction with the target membrane. In the context of the D614G spike and B.1.617.2 spike, we attached Cy5 fluorophore at position 836 at the FPPR region of the N terminus of the S2 domain, proximal to the fusion peptide, using genetic expansion code techniques (31). To facilitate fluorophore attachment, we incorporated one ncAA at position 836 through amber stop codon suppression (S*; Fig. S12). We then produced pseudovirions with the HIV core and SARS-CoV-2 S* for D614G S* as well as B.1.617.2 S* variants. The tagged S* was then labeled with Cy5-tetrazine by Diels-Alder cycloaddition (Materials and Methods). We chose this site to track the binding of fusion peptide in the S2 domain to the target membrane for fusion by following the anisotropy of the labeled fluorophore in real time. We find that the presence of PS lipid after triggering the virus with the addition of 500 mM Ca^2+^ and low pH led to a sudden increase in anisotropy, suggesting that PS lipid promotes the fusion peptide binding to the membrane (Fig. S8D). However, no change in anisotropy was observed in the absence of PS lipids in liposomes even after triggering the virus. This indicates that PS lipid promotes the spike binding to the target membrane. The anisotropy magnitude for both the D614G and B.1.617.2 was found to be significantly high, suggesting that PS lipid facilitates fusion peptide binding to the target membrane (Fig. S8D and E).

### Sequestering PS with LactC2 restricts SARS-CoV-2 spike-mediated cell-cell fusion

To understand the role of PS lipid in viral entry in host cells, we quantified the degree of internalization of SARS-CoV-2 spike pseudovirions into HEK293T cells expressing ACE2 and TMPRSS2, in the presence and absence of Lact-C2 expression. The pseudovirions were labeled with lipophilic dye DIO to coat the viral membrane. The HEK cells were infected with the pseudovirions and the number of virions inside the cell were counted using confocal imaging (Materials and Methods). For B.1.617.2, B.1.1.7, B.1.351, and D614G spike variants, we observed that the virus can internalize inside cell in the absence of Lact-C2, whereas the internalization is restricted to cells having Lact-C2 expression (Fig. S9). The spike pseudovirions were not able to enter cells in which Lact-C2 was expressed. DIC image showed the remarkable difference in viral uptake by cells with and without Lact-C2 expression (shown in red) (Fig. S9). Quantification of the number of viral internalizations revealed that B.1.617.2, B.1.1.7, B.1.351, and D614G spike pseudovirions were most readily able to infect cells in the absence of Lact-C2 expression, but Lact-C2 expression impeded the cellular entry (Fig. S9). This further indicates the important role of PS lipid in virus internalization and sequestering the PS lipid inside the cell inhibits the virus internalization for entry.

Next, to probe the role of PS lipid in spike-mediated cellular membrane fusion, we designed a cell-cell fusion assay between HEK293T/17 cell expressing the SARS-CoV-2 spike glycoprotein and GFP, and Vero-TMPRSS2 cell expressing mCherry (Fig. S10) (14). Incubation of 293T and Vero cells together resulted in cell-cell fusion between them, which was detected by the appearance of large, yellow-colored cells, resulting in the formation of a syncytia-like structure (Fig. S10). This cell-cell fusion assay was performed for four variants of SARS-CoV-2 spike protein: B.1.617.2, B.1.1.7, B.1.351, and D614G (Fig. S10). For all the spike variants, significant cell-cell fusion ability was observed, and Delta shows the highest fusion efficiency followed by B.1.1.7, B.1.351, and D614G spike strains. Strikingly, when Vero-TMPRSS2 cells were transfected with mRFP-Lact-C2 prior to the incubation with 293T cells having SARS-CoV-2 spike and GFP, the Lact-C2 expression stopped the cell-cell fusion with remarkable efficacy for all the spike variants and found the Lact-C2 expression in the target cell abrogates the SARS-CoV-2 spike-mediated cell-cell fusion ability (Fig. S10). We have also performed control experiments for the cell-cell fusion experiment, where HEK293T cells were expressing spike & GFP as donor cells, and Vero cells expressing PE lipid-binding protein (PEBP) with RFP were used as acceptor cells (Fig. S11). Vero cells expressing PEBP (red) were able to fuse to HEK293T cells expressing the B.1.617.2 spike and GFP (green). The fused yellow cells were indicative of the fact that PEBP expression does not affect the fusion activity of the spike to the host cell (Fig. S11). Whereas the expression of PS-binding protein Lact-C2 inhibits cell-cell fusion with significant efficacy (Fig. S10).

### Single-molecule FRET imaging of SARS-CoV-2 spike trimer and PS lipid interaction

**Fig 2:**
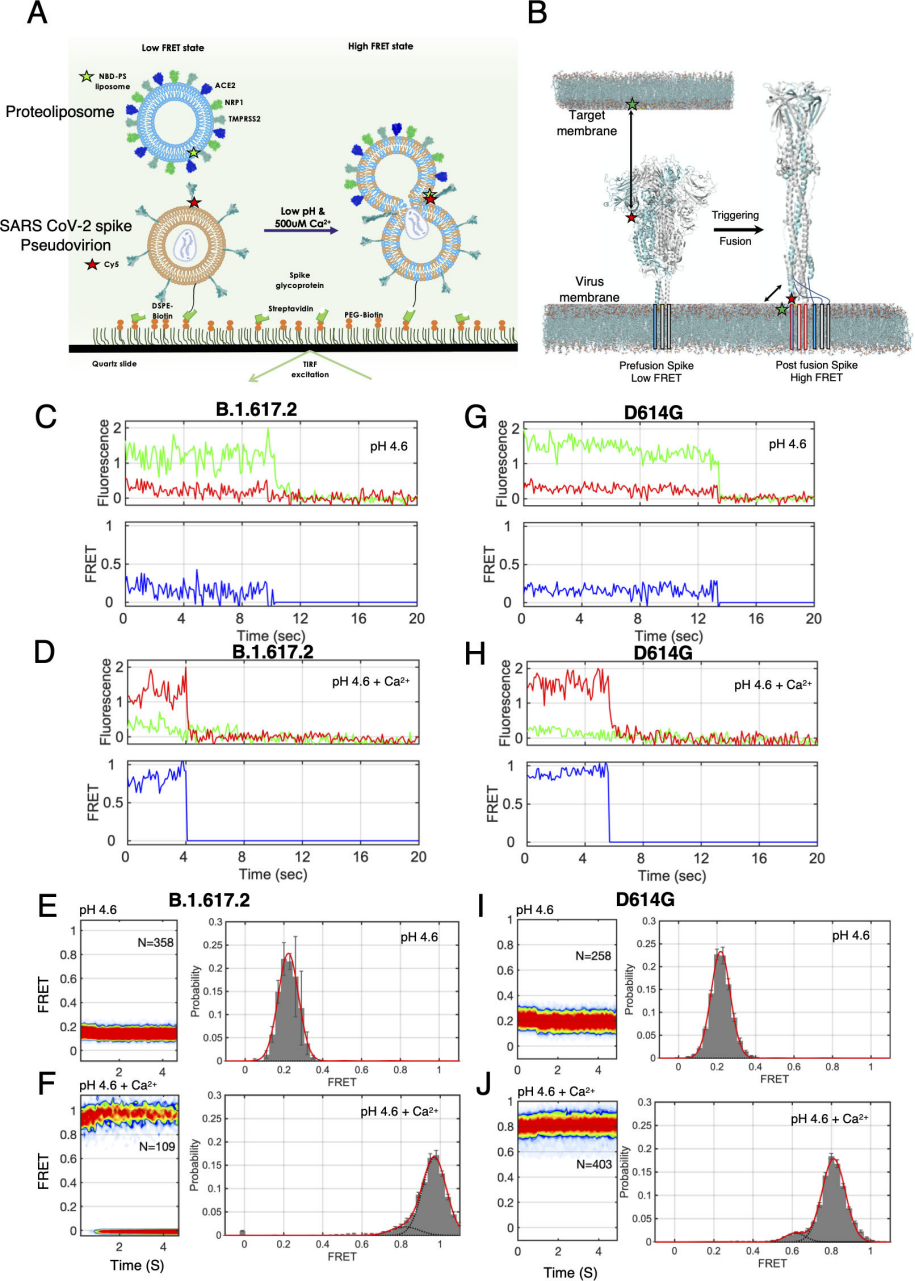
smFRET imaging SARS-CoV-2 spike trimer interaction with PS lipid in target membrane at fusion condition. (**A**) smFRET imaging assay design for probing the direct interaction between SARS-CoV-2 virus spike glycoprotein and PS lipid in target liposome at fusion condition. The PS lipid is present in the liposome and is labeled with NBD dye (donor) and the spike protein fusion domain is genetically labeled with Cy5 dye (acceptor). The pseudovirion was first immobilized on the quartz slide using biotin-streptavidin linkage, followed by the flowing of liposomes. smFRET imaging was performed at pH 4.6 and pH 4.6 and 500 μM Ca^2+^ conditions for B.1.617.2 spike and D614G spike pseudovirions. (**B**) Structural model of the prefusion Delta variants spike trimer with one protomer labeled with acceptor fluorophores (model based on PDB 7SBK; Materials and Methods). Unlabeled protomers within the trimer are shown in gray. The acceptor fluorophore is labeled at the fusion peptide proximal region of S2 (light blue), with the FL residing in a hydrophobic cleft at the protomer interface and contacting the neighboring protomer. The target bilayer membrane has donor fluorophore-labeled PS lipids. If the spike interacts with PS lipid during fusion, the change in FRET is anticipated from low FRET to high FRET state. (**C, D**) Representative fluorescence (donor, green; acceptor, red) and FRET trajectories (blue) obtained from a single B.1.617.2 spike of pseudovirion during *trans* interaction with PS lipid of liposome at low pH (**C**) or low pH with Ca^2+^ (**D**). (**E, F**) Contour plots (left) and smFRET histograms (right) were acquired immediately after exposure of B.1.617.2 spike pseudoviron to the low pH (**E**) or low pH with Ca^2+^ (**F**) shown for B.1.617.2 spike variants. (**G, H**) Representative fluorescence (donor, green; acceptor, red) and FRET trajectories (blue) were obtained from a single D614G spike of pseudovirions during *trans* interaction with PS lipid of liposome at low pH (**G**) or low pH with Ca^2+^ (**H**). (**I, J**) Contour plots (left) and smFRET histograms (right) were acquired immediately after exposure of D614G spike pseudovirons to the low pH (**I**) or low pH with Ca^2+^ (**J**) shown for D614G spike variants.

We next sought to directly visualize the interaction between SARS-CoV-2 spike trimers and PS lipids related to membrane fusion. For this purpose, we developed a smFRET imaging assay that could report on spike protein and PS lipid interaction at membrane fusion condition (Fig. 2A). In the context of the SARS-CoV-2 spike, we attached the first fluorophore at position 836 at the N terminus of S2 domain, proximal to the fusion peptide proximal region (FPPR). We attached the second fluorophore with the PS lipid present in the membrane of the target liposome.

We chose these sites to track the predicted conformational changes in S2 during membrane fusion that led to the displacement of the N terminus domain of the fusion loop with respect to the base of S2 and interaction with the target PS lipid to form post-fusion conformation. Structural data suggest a significant change in the distance (approximately 100 Å) between the fluorophores after the transition from the prefusion conformation to the post-fusion 6HB conformation, which we anticipated would yield an increase in FRET efficiency due to interaction between SARS-CoV-2 spike fusion peptide and PS lipid. We anticipated that if there is no interaction between the spike fusion peptide and PS lipid during fusion, it would provide low FRET, whereas if there is any interaction, it would provide high FRET efficiency (Fig. 2B).

To facilitate fluorophore attachment in the spike, we incorporated a trans-cyclooct-2-ene-L-lysine (TCO*) amino acids at position 836 (SARS-CoV-2 S*) through amber stop codon suppression (31, 32). We then formed pseudovirions with the HIV core and SARS-CoV-2 S* (Fig. S12). The pseudovirions were labeled with Cy5-tetrazine by Diels-Alder cycloaddition (Materials and Methods) (33, 34). Compared to wild-type D614G-S, D614G-S* and D614G-S*Cy5 maintained approximately 90% and 85% functionality in virus entry, respectively (Fig. S12B,D). The B.1.617.2-S* and B.1.617.2-S*-Cy5 are approximately 95% and 90% fusogenic relative to the B.1.617.2 spike, respectively (Fig. S12C,D).

The PS lipid of the target liposome was covalently linked with NBD dye. The NBD-PS lipid concentration in liposomes was maintained at 100 pM, to ensure that NBD-PS is present at a single-molecule level per liposome (Materials and Methods).

For smFRET imaging, spike pseudovirions were formed by diluting S* with an excess of wild-type S. The S* protomer was then labeled with Cy5. The ratio of S to S* was determined such that only a single fluorophore was seen in most virions (Fig. S12). The labeled virions were immobilized on quartz microscope slides followed by the addition of liposome having PS lipid and receptors (Fig. 2A). Next, triggering solution of low pH and Ca^2+^ (500 μM) was flown into the flow cell to initiate spike triggering for interaction with target membrane, which was imaged using total internal reflection fluorescence (TIRF) microscopy (Fig. 2A) (Materials and Methods).

### Fusion domain of spike S2 directly interacts with PS lipid in the target membrane by *trans* interaction at low pH and Ca^2+^

smFRET imaging for monitoring the direct interaction of PS lipid with the B.1.617.2 spike was carried out using liposomes having NBD-PS lipid as the donor and B.1.617.2 spike having Cy5 acceptor fluorophore. It has been established that ~500 μM Ca^2+^ and low pH are optimal for triggering spike-mediated fusion of SARS-CoV-2 (13). Here, we first imaged the interaction of B.1.617.2-S* and PS at pH 4.6, in the absence of Ca^2+^, conditions at which no fusion is observed. smFRET trajectories acquired from individual B.1.617.2 spike trimers on the virion surface showed a predominant low FRET state (Fig. 2C). The addition of 500 μM Ca^2+^ with pH 4.6 led to occupancy of a high FRET state (Fig. 2D).

These data indicate that Ca^2+^ binding of spike destabilizes the prefusion conformation and promotes conformational change in which the fusion domain of B.1.617.2 spike S2 subunit has moved to interact with the PS lipid of target liposome for fusion, which decreases the distance between the two fluorophores, giving rise to the remarkable transition from low FRET state (0.21 ± 0.08) to high FRET state (0.97 ± 0.08), as indicated by the smFRET histogram (Fig. 2E and F). To test whether this spike and PS interaction is conserved in other spike variants, we formed D614G-S* pseudovirion and performed smFRET imaging of interaction between the D614G-S* virion and PS lipid. D614G spike displayed stable low FRET at acidic pH 4.6 (Fig. 2G) and high FRET in the presence of Ca^2+^ and low pH (Fig. 2H). smFRET histogram suggests that the low FRET (0.20 ± 0.02) to high FRET (0.81 ± 0.07) state arises from a conformational change in spike that displaces the S2 fusion peptide into the target liposome to interact with PS lipid in the target membrane for fusion (Fig. 2I and J).

To test whether this spike and PS lipid interaction is pH and Ca^2+^ specific, we did smFRET imaging of spike and PS lipid interaction for both the B.1.617.2 and D614G variants at neutral pH with and without Ca^2+^. Both B.1.617.2-S* and D614G-S* displayed low FRET state in the presence of neutral pH with or without Ca^2+^ (Fig. S13), suggesting that no interaction is taking place between spike and PS lipid at neutral pH conditions. This confirms that the spike and PS lipid interaction for fusion is dependent on low pH and Ca^2+^.

Next, to confirm the lipid specificity of the spike for fusion, smFRET imaging was performed to probe the interaction between D614G S* and liposomes having NBD-PE lipid. PE lipid is also known to be transported in the cell surface. D614G spike displayed a low FRET state in the presence of PE lipid at both low pH and low pH with Ca^2+^ (Fig. S14A and B). Next, to see the role of other anionic lipid specificity of spike for fusion, smFRET imaging was performed to probe the interaction between D614G S* and liposome having NBD-PG lipid. D614G spike displayed a low FRET state in the presence of PG lipid at low pH, and in the presence of Ca^2+^, it majorly remains in a low FRET population with a minor population of high FRET state (Fig. S14C and D). This indicates that the spike has PS lipid specificity to a significant level in terms of interaction for promoting membrane fusion (Fig. S4).

### Intermediates states in spike-PS lipid interaction

**Fig 3:**
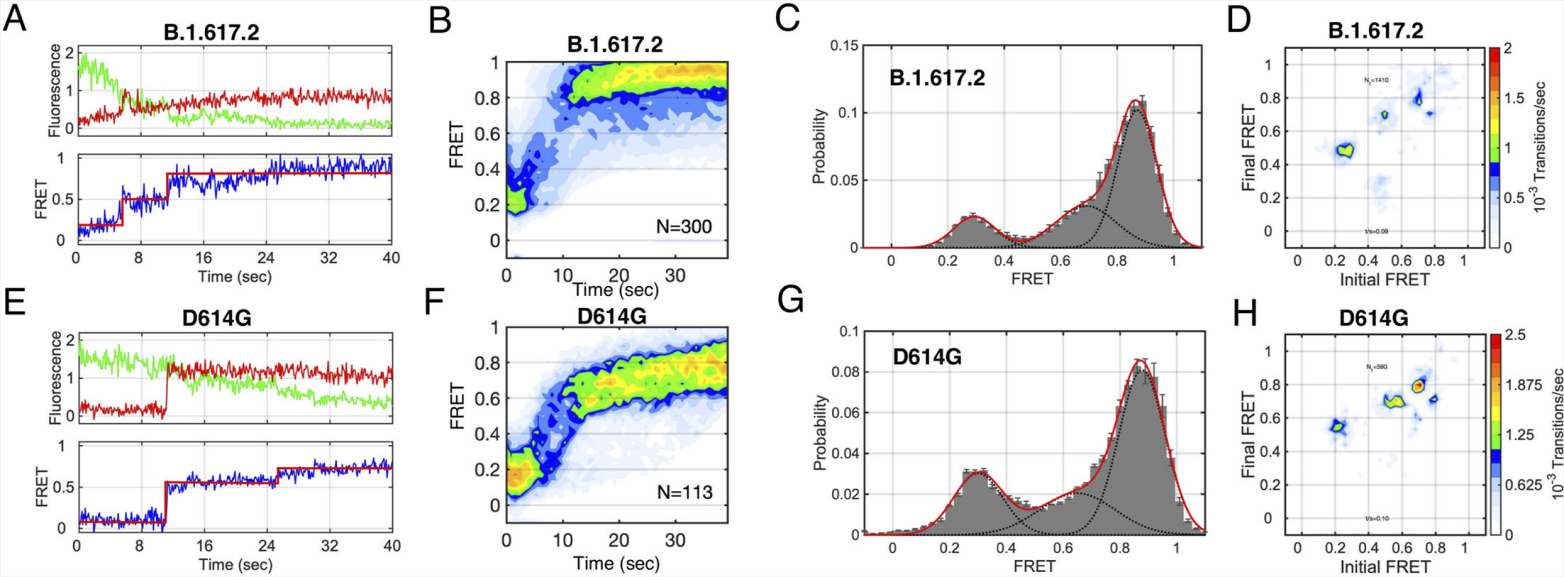
Fusion intermediates states during SARS-CoV-2 spike and PS-lipid interactions. (**A**) Representative fluorescence (donor, green; acceptor, red) and FRET trajectories (blue) obtained from a single B.1.617.2 S*-Cy5 protomer within an S trimer on the surface of a viral particle interacting with the PS-NBD lipid of target liposome. At each time point, FRET efficiency was calculated as the ratio of acceptor fluorescence intensity to total fluorescence intensity. (**B**) Contour plot of smFRET trajectories from a single B.1.617.2 S* and PS interaction synchronized to the transition out of low FRET. Transition through the intermediate-FRET state is observed in route to high FRET. (**C**) smFRET histogram displays the three FRET populations (**D**) and the transition density plots (TDP) display the distribution in initial and final FRET values for every transition observed from a single B.1.617.2 spike and PS interaction. The TDP indicates that trajectories predominantly transition from low to intermediate FRET and from intermediate to high FRET. No transitions have been observed directly between low and high FRET. This explains that fusion goes through an intermediate states. (**E, F, G, H**) Representative FRET trace (**E**), contour plot (**F**), smFRET histogram (**G**), and TDP plot (**H**) displayed for a single D614G S* pseudovirion and PS interaction at fusion condition.

To visualize the presence of any intermediate FRET state during spike fusion peptide and PS lipid interaction, we performed smFRET imaging at non-equilibrium conditions. smFRET imaging of the B.1.617.2-S* virion was started as soon as the triggering solution (pH 4.6 and 500 μM Ca^2+^) for fusion was flowed. Indeed, single-molecule trajectories show an intermediate FRET state during the transition from the low FRET state to the high FRET state (Fig. 3A). For B.1.617.2 spike-PS lipid interaction, we observed that low FRET state transits to high FRET state via an intermediate FRET state in an irreversible manner (Fig. 3B). smFRET histogram clearly shows the intermediate FRET state population along with low FRET and high FRET state populations (Fig. 3C). The asymmetric nature transition density plot defines the irreversible transition from the low FRET state to intermediate state and from intermediate state to the high FRET state (Fig. 3D). Similarly, for D614G-S*, we have observed intermediated FRET state on pathway from low FRET to high FRET state during fusion (Fig. 3E–H). The intermediate FRET states observed for both the B.1.617.2 and D614G spike might indicate the docking state of the fusion peptide with the target liposome for interaction with PS for the fusion reaction (Fig. 3C and G). The high FRET state defines the conformational state, where the fusion peptide is completely translocated into the target membrane. The irreversible transition from low FRET to intermediate FRET and from intermediate FRET to high FRET suggests the formation of stable post-fusion conformation of the spike protein.

### Spike S2 interacts with PS lipid of its viral membrane via *cis* interaction at low pH and Ca^2+^

**Fig 4:**
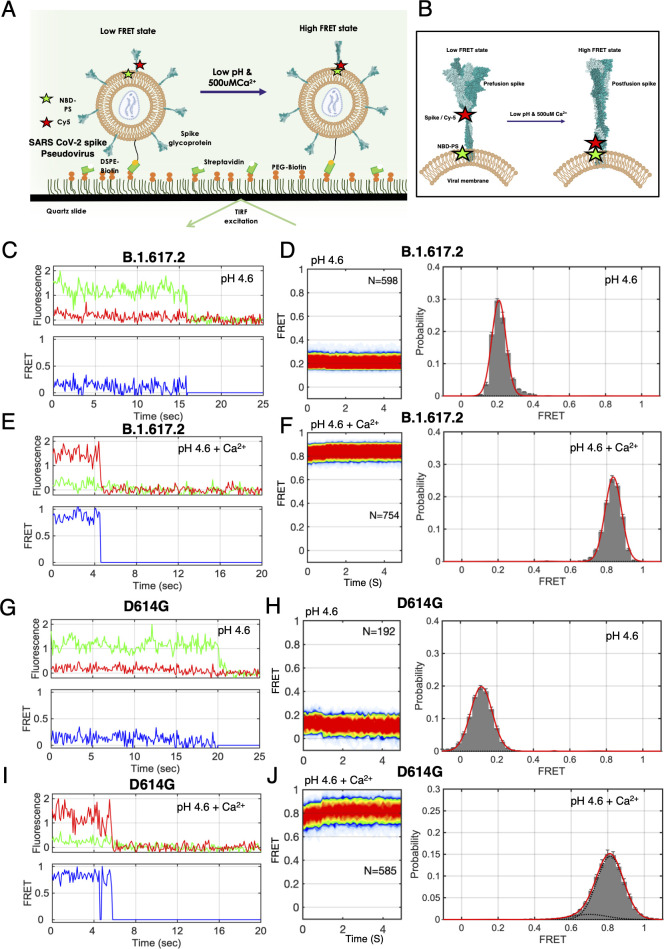
smFRET imaging *cis*-interaction between SARS-CoV-2 spike with PS lipid on the viral membrane at fusion condition. (**A**) smFRET imaging assay for *cis*-interaction between a single spike trimer of SARS-CoV-2 pseudovirion and PS lipid present in the viral membrane. The donor (NBD-PS) has been incorporated in the PS lipid of the viral membrane. The acceptor (Cy5) is tagged at the 836th position (FPPR residue) of the spike protein of the virion. Flowing of low pH and Ca^2+^ triggers the spike activation and interaction with PS lipids. (**B**) Model showing that a low FRET state would indicate less or no interaction between spike and PS lipid, whereas a high FRET state would indicate the spike interacting with the NBD-PS on the viral membrane. (**C, D**) Representative smFRET trace, contour plots (left), and smFRET histograms (right) for *cis* interaction between a single B.1.617.2 spike of pseudovirion and PS in the presence of low pH. (**E, F**) Representative smFRET trace, contour plots (left), and smFRET histograms (right) for *cis* interaction between a single B.1.617.2 spike of pseudovirion and PS in the presence of low pH with Ca^2+^ conditions. Low FRET is observed at low pH (**C**) and high FRET is observed at low pH with Ca^2+^ condition (**E**). (**G–J**) Representative smFRET trace, contour plots (left), and smFRET histograms (right) for *cis* interaction between a single D614G spike of pseudovirion and PS in the presence of low pH (**G, H**) or the presence of low pH with Ca^2+^ conditions (**I, J**). Low FRET is observed for D614G at low pH (**G**) and high FRET is observed at low pH with Ca^2+^ condition (**I**).

To understand whether spike can interact with the PS lipid present in the viral membrane, we designed smFRET assay for imaging *cis* interaction between the spike and PS lipid, where the Cy5-labeled spike pseudovirions were incubated with exogenous NBD-PS so that the PS can get incorporated in the viral membrane (Fig. 4A). We anticipated that if the fusion peptide of the spike would not be interacting with the PS on the viral membrane, it would yield a low FRET state, whereas if the spike does interact with own PS lipid on the viral membrane, it would provide high FRET state (Fig. 4B). We performed the smFRET imaging for B.1.617.2-S* virions having NBD-PS lipid on its membrane at pH 4.6 and observed the low FRET state (Fig. 4C and D). The addition of 500 μM Ca^2+^ at low pH shifts the FRET efficiency from a low FRET state to a high FRET state, indicating the notion that spike fusion peptide can interact with PS lipid of its membrane and may form a post-fusion-like conformation (Fig. 4E and F). Similar smFRET imaging experiments were carried out for imaging *cis* interaction between D614G-S* virions and PS lipid present on the virion membrane. D614G spike also shows a low FRET state (0.12 ± 0.02) at low pH (Fig. 4G and H). Whereas a high FRET state (0.81 ± 0.09) was observed at low pH with 500 μM Ca^2+^ condition (Fig. 4I and J). This suggests that low pH and Ca^2+^ trigger the conformational change in viral spike protein and in the absence of the target liposome, spike interacts with its own PS lipid and form post-fusion-like conformation. Therefore, the *cis* interaction is an alternative pathway through which spike fusion peptide may interact with PS of its viral membrane, but this interaction will inactivate the spike for membrane fusion.

Furthermore, to confirm the PS lipid presence on the surface of the SARS-CoV-2 virion membrane, we produced SEMN (spike, envelope, membrane, and nucleocapsid)-based virus-like particles (VLP) for Delta and D614G spikes (Materials and Methods). TIRF imaging of these VLP confirmed the presence of PS lipid on the SARS-CoV-2 virion membrane surface (Fig. S15).

### Molecular dynamics simulations of spike interaction with PS lipid and Ca^2+^

**Fig 5:**
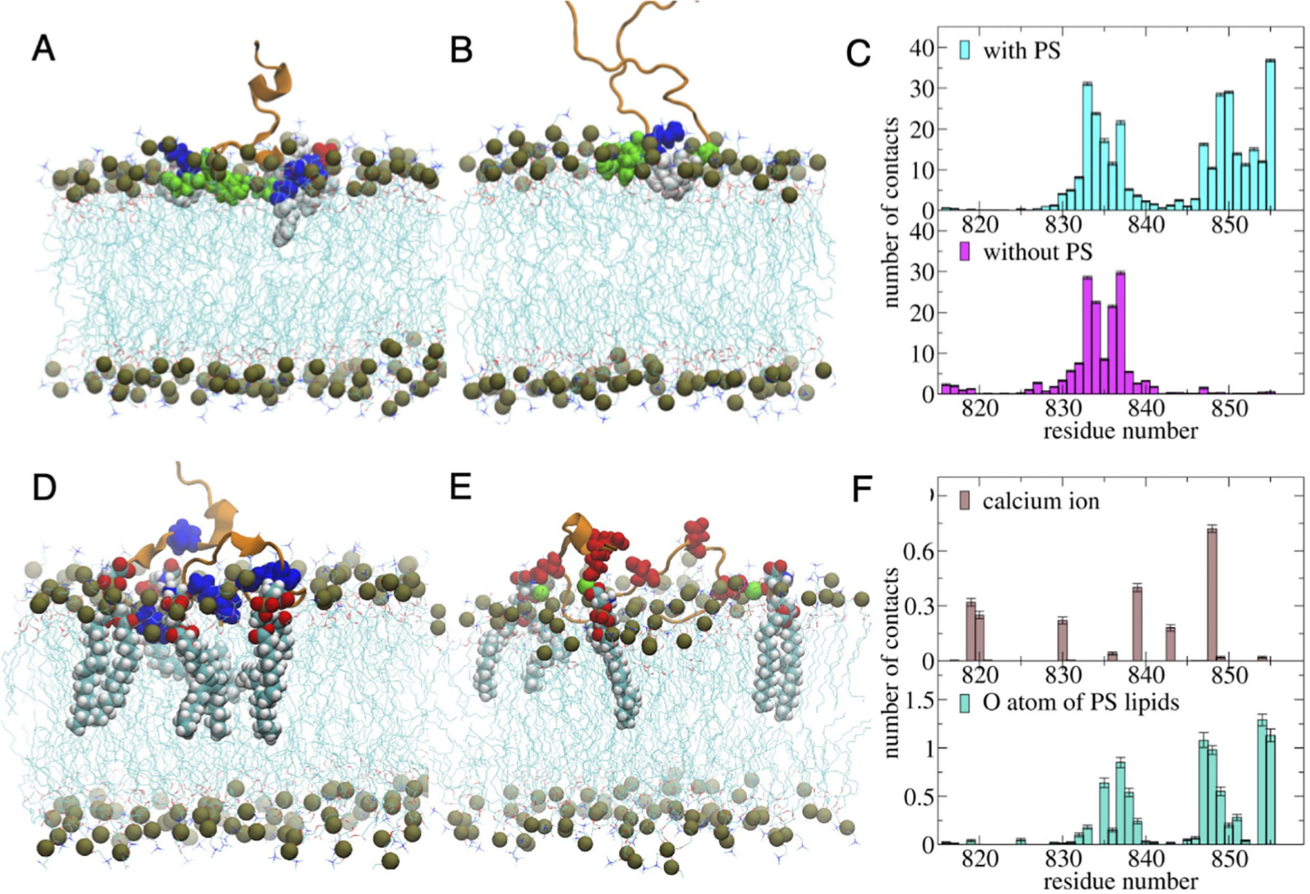
MD simulations of SARS-CoV-2 spike fusion peptide proximal regions (FPPR) within the lipid bilayers. Equilibrated structure of the S2 fusion peptide proximal residue (816–855 aa) adsorbed on the (**A**) DOPC/POPC/PS/CHOL bilayer and (**B**) DOPC/POPC/CHOL bilayer membrane, obtained after 1,000 ns long MD simulation. Peptide is shown in the cartoon (brown color) representation. The binding of the FPPR is significantly less in the absence of PS lipids in the membrane. (**C**) Number of lipid contacts with various residues of the FPPR in the presence (top) and absence (bottom) of PS lipids. (**D**) Representative snapshots show that some of the PS lipids (highlighted by VDW representation) preferentially bind with positively charged residues (blue) of the FPPR. (**E**) Representative snapshots show the Ca^2+^ (green) bridges between the negatively charged residues of the spike FPPR domain and PS lipids. (**F**) Time-averaged contact number between the FPPR residues and Ca^2+^ ions (top) and oxygen atoms of the PS lipids (bottom).

All atom molecular dynamics (MD) simulations were utilized to study the interaction of a single fusion peptide proximal region (FPPR) of S2 with a model lipid bilayer membrane consisting of 1,2-dioleoyl-sn-glycero-3-phosphocholine (DOPC), 1-palmitoyl-2-oleoyl-glycero-3-phosphocholine (POPC), 1-palmitoyl-2-oleoyl-sn-glycero-3-phospho-L-serine (PS), and cholesterol (CHL) lipids with a PC:CHL:PS stoichiometric composition of 4:4:1:1. Figure 5A presents the equilibrated structure of the fusion peptide proximal region (816–855 aa) from S2 domain of D614G spike (PDB: 7KRQ), in the membrane interface obtained after 1,000 ns long simulation. We have analyzed the structural characteristics of the bound state conformation of FPPR by computing the number of lipid atom contacts with each residue of fusion peptide (Fig. 5C, top). We find that FPPR is strongly interacting with the membrane surface through deep insertion of the hydrophobic residues, 833F, 834I, 849L, 850I, and 855F residues inside the membrane and strong adsorption of the polar/charged 835K, 836Q, 837Y, 847R, 848D, 853Q, and 854K residues on the membrane surface. To scrutinize the binding modes, we analyzed the number of contacts of the “O” atom of the PS lipids with each residue and found that the PS lipids are in contact with the positively charged residues 835K, 847R, and 854K and with a few neighbors of these residues of FPPR (Fig. 5F, bottom). We performed a control simulation in which the PS lipids were removed from the fusion peptide-bilayer membrane-bound configuration and the system was re-equilibrated (Fig. 5B). Figure 5B represents the membrane-peptide interface structure obtained after a 1,000 ns long simulation in the absence of any PS lipids in the membrane. We found that the FPPR is much weakly interacting with the membrane surface in the absence of PS lipids (Fig. 5B and C, bottom). The residues 847R, 848D, 849L, 850I, 851C, 852A, 853Q, 854K, 855F, and 835K, which were strongly bound to the membrane in the presence of PS lipids, were completely detached from the membrane surface in the absence of PS lipids (Fig. 5C). These results indicate that positively charged residues, 835K, 847R, and 854K, help bringing the fusion peptide closer to the membrane surface by interacting with the negatively charged PS lipids. Then the hydrophobic residues of the fusion peptide penetrate deep inside the hydrophobic membrane core and the polar residues bind with the hydrophilic lipid heads. We showed a representative binding mode of the positively charged 835K, 847R, and 854K residues with the PS lipids (Fig. 5D). We also found another binding mode of the fusion peptide with the PS lipids. In which, the negatively charged residues of the fusion peptide, 819E, 820D, 830D, 839D, and 848D engage in contact with Ca^2+^ through their backbone carbonyl or sidechain carboxylic end (Fig. 5F, top). These bound Ca^2+^ ions occasionally make bridges between these acidic amino acid residues, particularly the 839D and 848D of the fusion peptide and the PS lipid heads of the membrane (Fig. S16). Figure 5E presents a representative binding mode of the fusion peptide with the membrane through D-Ca^2+^-PS head bridge formation. Thus, spike fusion peptide can either directly interact with the PS lipids or via Ca^2+^ coordination pathway.

### Reversibility of S2 conformational change: *trans* interaction leads to membrane fusion

**Fig 6:**
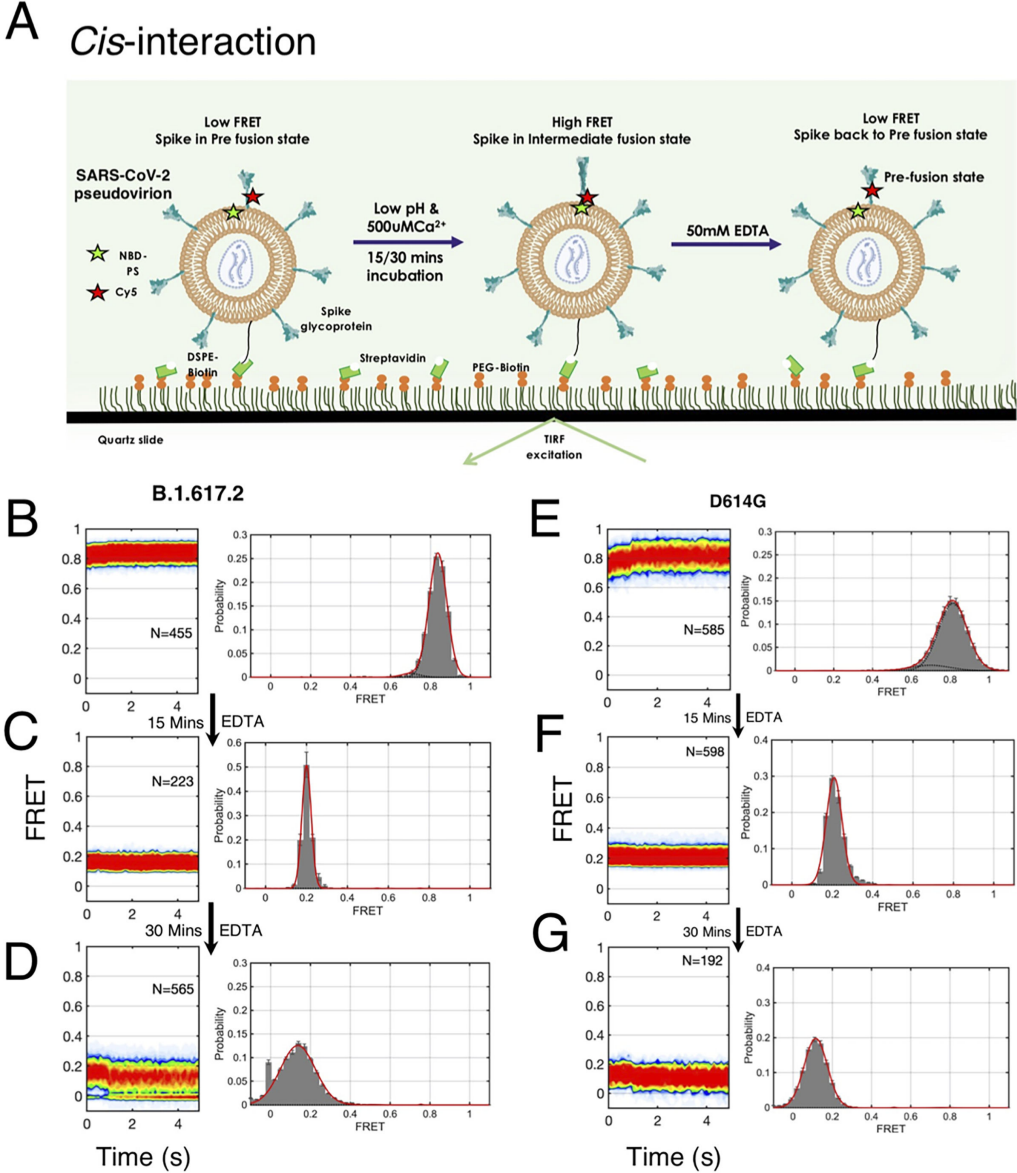
Reversible conformational dynamics of SARS-CoV-2 spike trimer of pseudovirion during *cis* interaction, in the absence of target membrane. (**A**) Single-molecule assay design for smFRET imaging the conformational changes of the spike during *cis* interaction with the PS lipid, present in the viral membrane. B.1.617.2 or D614G spike pseudovirions were genetically labeled with Cy5 fluorophore at the fusion peptide proximal region (FPPR) of the S2 domain of spike protein. The NBD fluorophore-labeled PS lipid was incorporated into the pseudovirion membrane. To follow the reversible conformational change of spike, first, dual fluorophore-labeled pseudoviron was immobilized in the quartz slide, second, low pH and Ca^2+^ solution were flown into the flow cell to trigger spike fusion, followed by flowing of EDTA (50 mM) solution at the indicated time delay. smFRET imaging of B.1.617.2 or D614G spike pseudovirion was acquired during fusion activation and after EDTA treatment at the indicated time duration. (**B, C, D**) Contour plots (left panel) and FRET histograms (right panel) during *cis* interaction for the B.1.617.2 spike pseudovirions and PS lipid. (**B**) Low pH and Ca^2+^ trigger the B.1.617.2 spike and PS interaction as indicated by the high FRET state. (**C**) The addition of EDTA after 15 minutes converts the high FRET state to the low FRET state, indicating that the high FRET state obtained in *cis* interaction is a reversible state and not a post-fusion state. (**D**) The addition of EDTA after 30 minutes shows the conversion of a high FRET state to a low FRET state. (**E, F, G**) Contour plots (left panel) and FRET histograms (right panel) during smFRET imaging *cis* interaction for the D614G spike pseudovirions and PS lipid. (**E**) Low pH and Ca^2+^ trigger the D614G spike and PS interaction as indicated by the high FRET state. The addition of EDTA after 15 minutes (**F**) or 30 minutes (**G**) completely converts the high FRET state to the low FRET state, indicating the reversibility nature of spike conformational change.

To test the reversibility in S2 dynamics in *cis* interaction, we designed a smFRET imaging assay in which we incubated the B.1.617.2-S* pseudovirion with NBD-PS lipid and exposed with low pH 4.6 and Ca^2+^ (500 μM) for 15 minutes before sequestering the Ca^2+^ with EDTA (Fig. 6A). To our surprise, the high FRET state obtained due to *cis* interaction between the Delta spike S2 fusion domain and the PS lipid of viral membrane fully returned to the low FRET state after sequestering the Ca^2+^ (Fig. 6B through D; Fig. S17). Sequestering the Ca^2+^ completely restored the prefusion low FRET state of the S2 domain (Fig. 6C and D; Fig. S17). Similarly, complete reversibility from high FRET to a low FRET state was observed for the D614G spike S2 domain (Fig. 6E–G). This indicates that the high FRET state obtained during the *cis* interaction is a reversible conformational state of S2 and it is distinct from the irreversible high FRET state seen for *trans* interaction. Therefore, if the fusion peptide gets trapped by interaction with its own viral PS lipid, the S2 domain is capable of reversible conformational change to the prefusion state to restore its functionality.

**Fig 7:**
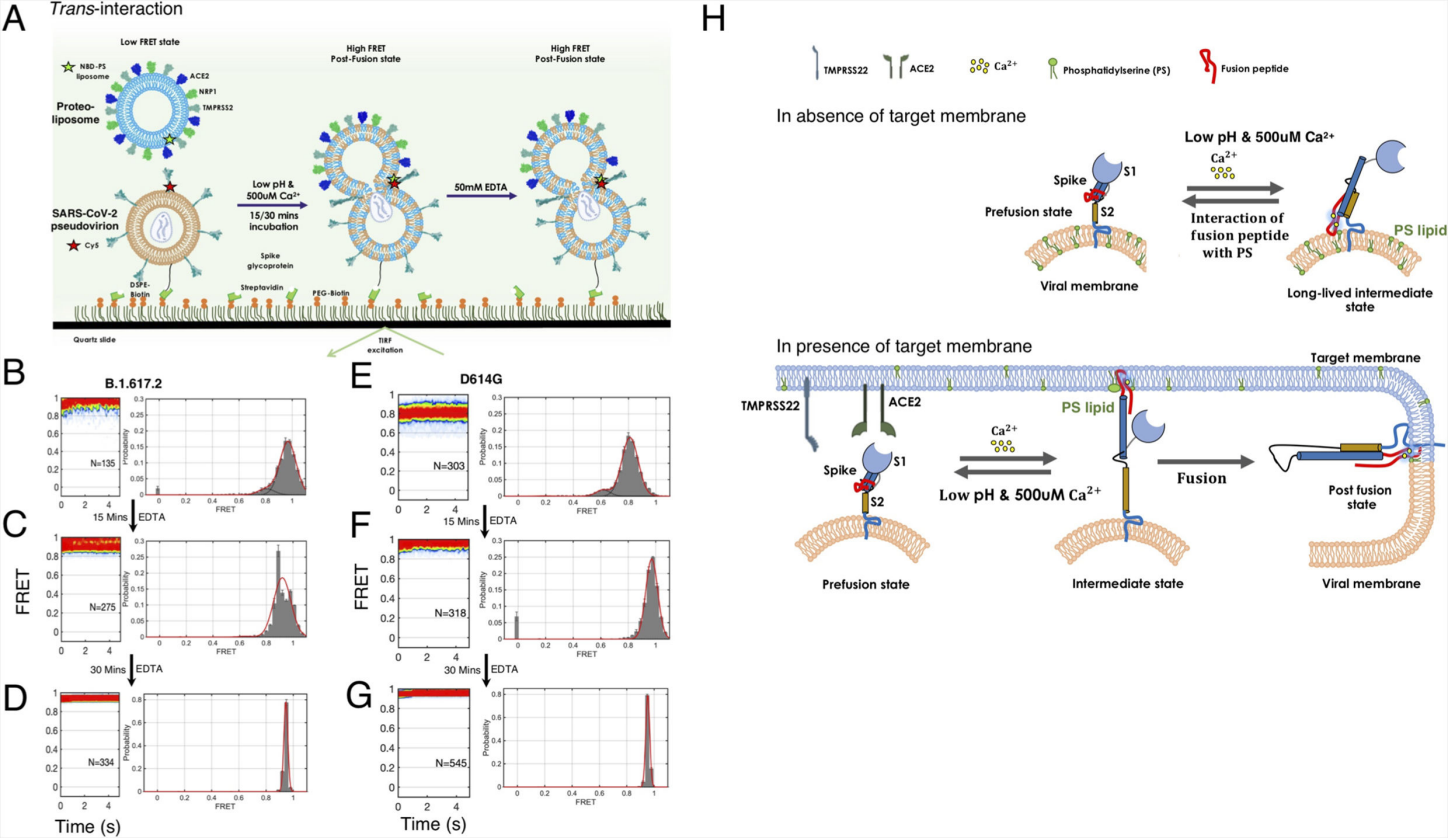
*trans* interaction of SARS-CoV-2 spike trimer of pseudovirion with PS lipid on target membrane promotes membrane fusion. (**A**) smFRET imaging assay design for probing the irreversibility nature of SARS-CoV-2 spike conformation during *trans* interaction with the PS lipid, for formation of post-fusion conformation. B.1.617.2 or D614G spike pseudovirions were genetically labeled with Cy5 fluorophore at the fusion peptide proximal region (FPPR) of the S2 domain of spike protein. The NBD fluorophore-labeled PS lipid was incorporated into the target liposomal membrane. To follow the *trans* interaction and conformational change of spike, first, acceptor fluorophore-labeled pseudovirons were immobilized in the quartz slide followed by donor-labeled PS-liposome was flown to form virion and liposome complex, second, low pH and Ca^2+^ solution was flown into the flow cell to trigger spike fusion, followed by flowing of EDTA (50 mM) solution at the indicated time delay. smFRET imaging of B.1.617.2 or D614G spike pseudovirion was acquired during fusion activation and after EDTA treatment at indicated time durations. (**B, C, D**) Contour plots (left panel) and FRET histograms (right panel) for trans-interaction for the B.1.617.2 spike pseudovirions during fusion activation (**B**) and after flowing of EDTA solution at 15-minute (**C**) or 30-minute intervals (**D**)**.** Low pH and Ca^2+^ trigger the spike and PS interaction as indicated by the high FRET state. The addition of EDTA does not affect the high FRET state of spike conformation, indicating the obtained high FRET state as an irreversible post-fusion state. Likewise, (**E, F, G**) Contour plots (left panel) and FRET histograms (right panel) for *trans* interaction for the D614G spike pseudovirions with PS lipid during fusion activation (**E**) and after flowing of EDTA solution at 15-minute (**F**) or 30-minute intervals (**G**). The high FRET state is maintained after the addition of EDTA, indicating the irreversible nature of spike post-fusion conformation. (**H**) PS lipid-dependent spike fusion model: smFRET analysis suggests that the SARS-CoV-2 spike acts as a reversible fusion machine. The spike trimers interact with the PS lipids of the target membrane in the presence of Ca^2+^ and low pH to promote membrane fusion. The spike has evolved in such a way that if it happens to interact with the PS lipids of its viral membrane then it can reversibly convert back into the pre-fusion state. The PS-dependent spike fusion is conserved across all the SARS-CoV-2 spike variants.

We next asked whether S2 could return from the high FRET state after transient exposure with low pH and Ca^2+^ during *trans* interaction with the target membrane. We performed smFRET imaging by exposing the B.1.617.2-S* virion and NBD-PS liposome mixture to pH 4.6 and Ca^2+^ for 15 minutes before sequestering the Ca^2+^ with EDTA (Fig. 7A). There was no change in the high FRET state even with sequestering the calcium, suggesting that the high FRET state was entirely irreversible with the additional inclusion of Ca^2+^ (Fig. 7B through D; Fig. S17). Thus, virion incubation with PS liposome at acidic pH and Ca^2+^ induced the transition of the S2 domain of B.1.617.2 spike to a conformation that could not be reversed by sequestering the Ca^2+^ (Fig. 7C and D; Fig. S17). Similarly, D614G-S* pseudovirion and PS liposome mixture were exposed to pH 4.6 and Ca^2+^ for 15 minutes before sequestering the Ca^2+^ with EDTA (Fig. 7E). Likewise, no change in the high FRET state was observed for D614G spike even with sequestering the Ca^2+^, suggesting that the high FRET state acquired due to *trans* interaction between the spike S2 domain and PS lipid led to a formation of irreversible conformation (Fig. 7F and G). Therefore, the irreversible high FRET data indicate the formation of post-fusion conformation of spike due to *trans* interaction between the S2 domain and PS lipid of the target liposome.

## DISCUSSION

Our study provides direct observations of conformational changes in intact trimeric SARS-CoV-2 S fusion protein on the surface of the virion, during interaction with PS lipid molecule, related to membrane fusion. Our results suggest a model of spike-mediated membrane fusion with essential roles of PS lipid, acidic pH, receptor binding, and endosomal Ca^2+^ (Fig. 7H). According to this model, spike S2 domain and PS lipid interaction are essential for the promotion of membrane fusion. Acidic pH, receptor binding, and Ca^2+^ promote the spike and PS lipid interaction (35). It has been previously established that TMPRSS2-mediated SARS-CoV-2 spike cleavage activates the fusion at neutral pH (10–12). Our study demonstrates that the acidic pH and Ca^2+^ catalyze the spike-mediated fusion reaction pathway in a PS lipid-dependent manner.

It is equally possible that the spike and PS interaction can work either in the same side (*cis*) or the *trans* side after binding to ACE2 and activating protease TMPRSS2. Prior to membrane fusion, the spike S2 domain mostly remains in the prefusion conformations. Given the proximity of the fluorophore attachment site at the fusion peptide proximal region of the S2 domain and at the PS lipid, the FRET states observed here likely reflect conformations in which the S2 fusion peptide adopts distinct positions. First, the low FRET state is consistent with the prefusion conformation of the spike in which the fusion peptide in S2 is far from the PS lipid present in the membrane. Second, an intermediate FRET state reflects a rearrangement of the S2 domain and docking of fusion peptide into the lipid membrane after the large-scale displacement predicted by the canonical model of class-I viral membrane fusion (2, 3). Third, the spike S2 domain has access to a high FRET state in which the S2 fusion peptide has moved closer to the PS lipid of the viral or target membrane. When the spike and PS interaction is *cis* in nature, our observations demonstrate that acidic pH and Ca^2+^ directly initiate conformational remodeling of the spike by shifting the equilibrium in favor of the reversible high FRET conformation. Remarkably, the low FRET prefusion conformation is reformed upon the removal of Ca^2+^. We anticipated that if the spike S2 and PS interaction led to a post-fusion conformation, it would yield an irreversible high FRET because of the thermodynamic stability of similar envelope fusion protein in this conformation. Therefore, the reversible conformational change in S2 suggests that the *cis* interaction of spike and PS lipids do not promote the transition of the S2 domain to the post-fusion six-helix bundle (6-HB) conformation. Rather, we speculate that the reversible high FRET state is an intermediate conformation that is on the pathway to the post-fusion conformation. This intermediate conformation is a kinetically stable state, suggesting a structure in which the fusion peptide position is localized near the viral membrane, and it may interact with the PS lipid of the own viral membrane.

By contrast, when the spike and PS interaction is *trans* in nature, the presence of PS lipid in target liposome membrane enables S2 domain transition to an irreversible high FRET conformation. Thus, the S1 assists with allosterically regulating the timing of S2 conformational changes, ensuring that S2 does not prematurely trigger prior to the arrival of the virion in a late endosome that contains low pH and Ca^2+^. The receptor engagement with S1 followed by the triggering of the S2 domain by low pH and Ca^2+^ led to conformational change in S2 domain and binding of fusion peptide with the PS lipid in the target membrane via *trans* interaction. This eventually led to the formation of an irreversible high FRET post-fusion state and promoted membrane fusion. Therefore, the spike has evolved in such a way that it can discriminate between the *cis* interaction with PS lipid in viral membrane and the *trans* interaction with PS lipid in the target membrane. If the *trans* interaction fails to happen, then the reversibility nature of conformational change in the spike due to *cis* interaction saves the spike from inactivation and it allows the spike to attain prefusion conformation so that the virion could utilize it again for the fusion reaction.

In summary, we have shown that PS lipid is an important cofactor for SARS-CoV-2 membrane fusion for entry and SARS-CoV-2 spike is a reversible fusion machine. These spike and PS lipid interactions are mediated by low pH, Ca^2+^, and receptor binding, which regulate the function of spike during membrane fusion. The mechanisms of this regulation are diverse, occurring through both long-range allosteric and direct interactions with the spike fusion domain and PS. The exposure of spike RBD may facilitate the binding with all receptors, but for triggering the membrane fusion, the spike fusion domain requires PS lipid in the target membrane. The low pH and Ca^2+^ binding trigger the conformational changes in the spike for interaction with PS lipid in the target membrane for fusion. Therefore, PS signaling is important for SARS-CoV-2 entry and the ability to target the PS-binding site in the spike may provide a better route for COVID-19 therapies.

## MATERIALS AND METHODS

### Production of DiO-labeled lentiviral pseudovirions having SARS-CoV-2 variant spikes

Plasmids encoding different SARS-CoV-2 spike variants (D614G/B.1.351/B.1.1.7/B.1.617.2) and a plasmid encoding HIV-1 GagPol were transfected in HEK293T/17 cells using lipofectamine (Invitrogen), in a ratio of 5:5 of spike:gagPol (13). All the plasmids encoding spike variants were obtained from Stephan Pohlmann laboratory (University of Gottingen) (6, 7). The plasmid encoding HIV-1 gagpol was a gift from Walther Mothes lab (Yale University) (32).

HEK293T/17 cells were maintained in DMEM (GIBCO), supplemented with 10% FBS (GIBCO), 2 mM L-glutamine (GIBCO), and 100 U/mL penicillin/streptomycin (GIBCO) at 37°C, with 5% CO_2_, and transfected at 70–75% confluency. At 72 hours post-transfection, the supernatant was collected and filtered through a 0.45 μm filter to remove cell debris. Viruses were concentrated in a 10% sucrose cushion by ultra-centrifugation at 25,000 × *g* for 2 hours. The concentrated pseudovirions were then labeled with 20 uM DiO (Invitrogen) for 2 hours at room temperature in rotation. The labeled pseudovirions were purified using a 6–30% OptiPrep gradient (Sigma-Aldrich) by centrifugation at 35,000 × *g* for 1 hour. The labeled fractions having the virions were aliquoted and stored at −80°C for further use.

### Cloning, expression, and purification of mRFP-Lact-C2

Lactadherin-C2 (Lact-C2) tagged to mRFP was amplified from pCMV-mRFP-Lact-C2 plasmid (Addgene) (28). It was then cloned between NdeI and XhoI sites in the bacterial expression vector pET-28a(+). The clone was then used to transform BL21(DE3) cells according to the manufacturer’s protocol (Invitrogen). Cells were grown in LB medium at 37°C until the O.D. reached around 0.66. Induction was done using 0.75 M IPTG, at 16°C overnight. Cells were lysed in the presence of PMSF (Sigma) by sonication. After centrifugation at 18,000 × *g* for 1 hour, the protein was first affinity purified using Ni-Sepharose beads (GE Healthcare). Lact-C2-fused mRFP has a thrombin cleavage site before the 6×His tag at the N terminus. The His tagged domain was cleaved using thrombin (1% [wt/wt]). The sample was further purified using size exclusion chromatography using Superdex 200 increase 10/300 Gl column (GE Healthcare). Samples were exchanged into a buffer having 20 mM HEPES and 50 mM NaCl for final storage. Stock solutions of the protein were stored with ~16% glycerol at −80°C.

### Cell-cell fusion assay

Two different cell lines, HEK293T/17 and Vero-TMPRSS2, were used for cell-cell fusion assay (14). HEK293T/17 cells were transfected with the spike plasmid of the respective variant along with GFP in a ratio of 2:2. Vero-TMPRSS2 cells were transfected with either mCherry or mRFP-Lact-C2. At 24–30 hours post-transfection, cells were seeded at around 1 million densities on 0.01% poly-D-lysine (Sigma) and 4 μg/mL fibronectin (Sigma) pre-treated glass bottom confocal dishes (SPL Lifesciences). HEK293T and Vero-TMPRSS2 cells were mixed in a ratio of 1:1 in DMEM supplemented with 10% FBS and Pen-Strep. The cells were allowed to attach to the confocal dishes for 24 hours, following which confocal imaging was performed (14). Images were acquired using a Zeiss LSM710NLO confocal microscope under a 20× objective, with a motorized XY stage for housing samples within a CO_2_ enclosure and temperature-controlled platform. For the green channel (GFP), a multi-line argon laser source was used; and a diode pump solid-state laser source was used for the red channel (mCherry/mRFP). To avoid any crosstalk between the channels, the laser power and bandwidths were selected accordingly. The images acquired were processed in the Zen (Blue) software suite from ZEISS. To quantify cell-cell fusion, Mander’s coefficient was calculated using the JACoP plugin in ImageJ. Five different areas were used for calculating the mean ± SEM of the Mander’s coefficient for each case.

### Virus internalization assay

HEK293T/17 cells were transfected with full-length ACE2, full-length TMPRSS2, and mRFP-Lact-C2 plasmids in a ratio of 2:2:2 (in µg). At 24 hours post-transfection, the transfected cells were transferred to pre-coated confocal dishes (coated with poly-D-lysine) at a final cell density of around 1 million. The cells were allowed to attach to the dish for 24 hours, following which, the cells were infected with 100 μl DiO-labeled pseudovirions. Cells were incubated with the virus at 4°C for 10 minutes to aid greater attachment of the virus to the cells. The plates were then incubated for 2 hours at 37°C to allow the viruses to enter inside the cells. After 2 hours, the plates were washed twice with PBS, and phenol red-free DMEM was added. Confocal data were recorded using LSM 780, Axio Observer microscope (Zeiss) using either a Plan-Apochromat 40×/1.4 Oil DIC M27 or a Plan-Apochromat 63×/1.4 Oil DIC M27 objective. mRFP-Lact-C2 was excited using a 561 nm laser, and DiO-labeled virions were excited with a 488 nm laser. Multiple z-stacks spanning the entire cell volume with z-planes spaced 300 nm apart were acquired.

### Virus entry inhibition assay

HEK293T/17 cells were transfected with full-length ACE2 and full-length TMPRSS2 plasmids in a ratio of 2:2. The cells were seeded in confocal dishes 24 hours post-transfection. The cells were then incubated with 100 nM mRFP-Lact-C2 protein diluted in DMEM at 37°C for 45 minutes. For a control set, the cells were incubated with 500 nM Lysotracker Red (Invitrogen) for 10 minutes, following which the cells were washed twice with PBS. The cells that were incubated with 100 μl DiO-labeled virions were then added to the cells and the plates were incubated for 2 hours at 37°C. After 2 hours, the plates were washed twice with PBS buffer, and phenol red-free DMEM was added. Images were acquired in the set-up and the specifications described above at 63× magnification by a 1.4 NA oil-based objective. Analysis was performed in Zen Blue and ImageJ. The number of virions was counted using automatic counting in ImageJ by defining a threshold.

### Liposome preparation

Liposomes with phosphatidylserine (PS) were prepared in a 4:4:0.5:0.1:2 ratio of 1,2, dioleoyl-sn-glycero-3-phosphocholine (DOPC; Avanti Polar Lipids), 1-palmitoyl-2-oleoyl-glycero-3-phosphocholine (POPC; Avanti Polar Lipids), phosphatidylserine (PS; Avanti Polar Lipids), Ni-NTA DGS lipid (Avanti Polar Lipids), and cholesterol (Avanti Polar Lipids) (13, 31, 32). Briefly, the lipids were mixed in chloroform, which was evaporated under a stream of argon gas. The dried lipid film was resuspended in HNE buffer (5 mM HEPES, 145 mM NaCl; pH 7.5), which was followed by five freeze-thaw cycles. Liposomes of around 100 nm were obtained by extruding the homogenized aqueous lipid suspension through a 100 nm polycarbonate membrane filter. The freshly prepared liposomes were coated with histidine-tagged hACE2, NRP1-b1, and hTMPRSS2 in a ratio of 1:1:1 at 37°C for at least 1 hour in rotation (13). Liposomes without phosphatidylserine (PS) were prepared in a 4:4:0.1:2 ratio of 1,2, dioleoyl-sn-glycero-3-phosphocholine (DOPC; Avanti Polar Lipids), 1-palmitoyl-2-oleoyl-glycero-3-phosphocholine (POPC; Avanti Polar Lipids), Ni-NTA DGS lipid (Avanti Polar Lipids) and cholesterol (Avanti Polar Lipids).

Liposomes for smFRET imaging were prepared by the protocol described above. Liposomes having NBD-PS were prepared in a 4:4:0.000625:0.1:2 ratio of 1,2,dioleoyl-sn-glycero-3-phosphocholine (DOPC; Avanti Polar Lipids), 1-palmitoyl-2-oleoyl-glycero-3-phosphocholine (POPC; Avanti Polar Lipids), 1,2-dipalmitoyl-*sn*-glycero-3-phosphotidylserine-N-(7-nitro-2–1,3-benzoxadiazol-4-yl) (NBD PS; Avanti Polar Lipids, cat# 810194), Ni-NTA DGS lipid (Avanti Polar Lipids), and cholesterol (Avanti Polar Lipids). Liposomes having NBD-PE were prepared in a 4:4:0.000625:0.1:2 ratio of 1,2,dioleoyl-sn-glycero-3-phosphocholine (DOPC; Avanti Polar Lipids), 1-palmitoyl-2-oleoyl-glycero-3-phosphocholine (POPC; Avanti Polar Lipids), 1,2-dipalmitoyl-*sn*-glycero-3-phosphotidylethanolamine-N-(7-nitro-2–1,3-benzoxadiazol-4-yl) (NBD PE; Avanti Polar Lipids, cat# 810155), Ni-NTA DGS (Avanti Polar Lipids), and cholesterol (Avanti Polar Lipids). The liposomes were coated with recombinant hACE2, NRP1-b1 and hTMPRSS2 with the following molar concentration of lipids and proteins: 1,2-dioleoyl-sn-glycero-3-phosphocholine (DOPC; Avanti Polar Lipids): 31.8 mM, 1-palmitoyl-2-oleoyl-glycero-3-phosphocholine (POPC; Avanti Polar Lipids): 32.9 mM, phosphatidylserine (PS; Avanti Polar Lipids): 6 mM, Ni-NTA DGS lipid (Avanti Polar Lipids): 5 mM, cholesterol (Avanti Polar Lipids): 25 mM, recombinant ACE2: 1.8 µM, recombinant NRP1: 1.8 µM, and recombinant TMPRSS2: 1.8 µM.

### Virus-liposome lipid mixing assay

The virus-liposome lipid mixing assay has been described extensively in our previous study (13). Briefly, DiO-labeled pseudovirions with either WT spike or D614G spike were combined with proteo-liposomes (either with or without PS) coated with hACE2, NRP1-b1, and hTMPRSS2. Buffer solution was added in a stop-flow manner to adjust desired pH conditions and DiO fluorescence was followed in a time-based manner. DiO was excited at 488 nm and fluorescence was detected at 515 nm at every second’s intervals for 20 minutes in a QuantaMaster fluorescence spectrophotometer 8450 (Horiba). All the fusion experiments were performed at 37°C by a rapid Peltier temperature-controlled sample holder. Data were acquired with the FelixGx software provided by the manufacturer. All the data were analyzed and plotted using MATLAB.

For Lact-C2 titration, different concentrations of Lact-C2, ranging from 0 pM to 25 nM were used. Liposomes having Ni-NTA DGS were incubated with the designated concentration of Lact-C2 (possessing His-tag) for 60 minutes in rotation. The liposomes were also coated with ACE2, NRP1, and TMPRSS2 along with Lact-C2. The fusion reaction was carried out in the same manner described above.

### Virus fusogenicity assay

The fusogenicity of the pseudotyped virions was tested by a β-lactamase (BlaM)-based enzymatic assay (LiveBlazer FRET B/G kit, Invitrogen) (30, 32). The pseudoviruses were formed as described above, but with an additional plasmid encoding BlaM, fused to the HIV-1 Vpr protein. Viruses were collected and concentrated as described above and resuspended in phenol red-free DMEM (GIBCO), supplemented with 10% FBS (GIBCO), 2 mM L-glutamine (GIBCO), and 100 U/ml penicillin/streptomycin (GIBCO). Vero-TMPRSS2 cells were seeded in 96-well plates and incubated with either Lact-C2 or exogenous PS. Lact-C2 diluted in DMEM was used at a final concentration of 100 nM, and PS in chloroform diluted in MilliQ water was used at a final concentration of 2.5 μM. The virions were then used for infecting HEK293T/17 cells. Spinoculation was done at 3,700 rpm at 4°C. Unbound viruses were removed by washing with HBSS (GIBCO) and resuspending the cells in fresh phenol red-free DMEM. The plate was then incubated at 37°C for 90 minutes to permit viral entry. Cells were then loaded with the substrate, CCF4-AM fluorophore, in the presence of probenecid at a final concentration of 2.5 mM. The plate was incubated overnight at 11°C. The cleavage of CCF4-AM by BlaM was detected using a plate reader (Biotek). The fusogenicity of the virus was calculated as a ratio of blue to green emission.

### Incorporation of non-canonical amino acid in the SARS-CoV-2 spike (S*)

A single non-canonical amino acid (ncAA), TCO*, was incorporated into the S2 domain of the SARS-CoV-2 spike through amber stop codon suppression technology, as described previously (31, 32). TAG codon was introduced at position 836 of S2 near the fusion peptide proximal residue (FPPR). Translation to incorporate the ncAA was allowed to proceed through the UAG codons on the mRNA in the presence of an orthogonal tRNA (tRNA^Pyl^), which recognizes the UAG codon. A corresponding aminoacyl-tRNA synthetase (NESPylRS^AF^) was used which aminoacylates the suppressor tRNA with TCO*, facilitating its incorporation at the 836th position into S, forming S*. The efficiency of amber suppression is limited due to competition of the eukaryotic release factor 1 (eRF1) with tRNA^Pyl^. Expression of the dominant negative eRF1 E55D mutant increased amber suppression efficiency (36).

HEK293T cells were transfected with plasmids encoding the spike, TAG-mutated S*, NESPylRS^AF^/tRNA^Pyl^, eRF1 E55D, and HIV-1 Gag-Pol in a ratio of 2.5:2.5:2.5:1.5:5. The growth medium was supplemented with 500 μM TCO* ncAA (SiChem). At 72 hours post-transfection, the virus was harvested using a 0.45-μm syringe filter. The virions were concentrated in a 10% sucrose cushion by centrifugation at 25,000 × *g* at 4°C. The concentrated virions were labeled with DSPE-biotin for 30 minutes in rotation, following which 500 μM Cy5 was added. Cy5-labeled virions were purified in a 6%–30% Opti-Prep gradient by centrifugation at 35,000 × *g* for 1 hour. The fractions having the labeled virions were collected and used for smFRET imaging (Fig. S12).

For the *cis*-interaction assay, pseudovirions with S*-Cy5, and labeled with DSPE-biotin, were incubated with a final concentration of 0.6 nM NBD-PS for 1 hour in rotation so that NBD-PS is incorporated in the membrane of the virions. Labeled virions were then purified using 6%–30% Opti-Prep, as described above.

### smFRET imaging of SARS-CoV-2 virions and PS lipid interaction

smFRET imaging was done using a home-built prism-based TIRF microscope. Quartz slides passivated with polyethylene glycol (PEG) were used for imaging. Slides were coated with streptavidin to immobilize DSPE-biotin-tagged virions. Liposomes were flowed once the virions were immobilized on the slide. Unbound liposomes were removed by washing with 100 μl of triggering solution. The slides were mounted on an XY-piezo stage (Applied Scientific Instruments). An evanescent field was created by total internal reflection using a 488 nm solid-state laser (Coherent), at a power of 150 mW. Fluorescence emission was collected with a 60× water-immersion objective. Donor and acceptor emissions were separated using a dichroic filter and the emissions were collected using synchronized sCMOS cameras (Hamamatsu). Images were acquired at 5 frames/millisecond using custom-built MicroManager software. The imaging cocktail contained 50 mM Tris (pH 8.0) and 50 mM NaCl, along with triplet state quenchers (1 mM trolox, 1 mM cyclooctatetraene, 1 mM nitrobenzyl alcohol) and beta-mercaptoethanol (37, 38). The cocktail also included an enzymatic system for the removal of molecular oxygen, which included 2 mM protocatechuic acid (PCA) and 8 nM protocatechuate 3,4-deoxygenase (PCD) (37, 38). The buffers for different pHs were made using a combination of citric acid and disodium hydrogen phosphate, and the triplet state quenchers and oxygen scavengers were added to them.

For the *cis*-interaction assay, after immobilizing viruses on the PEGylated slide, the triggering solution (pH 4.6/pH 4.6 and 500 μM Ca^2+^/pH 7/pH 7 and 500 μM Ca^2+^) was flowed. Imaging was performed using the same conditions as described above. The conformation reversibility experiment involving EDTA was performed by incubating the virions with pH 4.6 and 500 μM Ca^2+^ for 15 minutes/30 minutes (as indicated) and then flowing 50 mM EDTA to chelate all the Ca^2+^ present in the surroundings. All the pH and EDTA solutions contained the components of the imaging cocktail to prevent quenching of the fluorophores.

### Viral membrane phosphatidylserine-Lact-C2 colocalization assay

Virus-like particles (VLPs) having spike, envelope, membrane, and nucleocapsid proteins (S-E-M-N) proteins were prepared by transfecting all the plasmids in HEK293T/17 cells in 5:5:5:5 ratio *(*13*).* VLPs with B.1.617.2 and D614G spike were produced. At 72 hours post-transfection, the VLPs were collected and concentrated using the steps described above. The virions were labeled with DiO and DSPE-biotin, and the labeled virions were separated from free dye using OptiPrep and density gradient centrifugation as mentioned before. The labeled VLPs were flowed on a pegylated quartz slide and incubated for 30 minutes, followed by recombinant Lact-C2 labeled with mRFP in the same channel (Fig. S15). The VLPs were incubated with Lact-C2 for 30 minutes to allow the Lact-C2 to bind to the viral membrane phosphatidylserine, if present. The channel was washed twice with 100 μL T50 buffer to remove unbound or excess Lact-C2. The slide was mounted on an XY-piezo stage. VLPs were imaged using a 488 nm blue laser, and a green laser was used for imaging Lact-C2. Images acquired were then analyzed and merged using ImageJ (39).

### MD simulations

Initial coordinates of the fusion peptide (S2) were taken from the protein data bank (PDB ID: 7KRQ). A single S2 fusion peptide proximal residue (816–855 aa) was placed on top of a lipid bilayer consisting of DOPC, POPC, PS lipids, and cholesterols. The membrane-protein complex was then solvated using a TIP3P water box (40). An appropriate number of Ca^2+^ and Cl^−^ was added to maintain charge neutrality. The built structure was then energy minimized using the steepest descent and conjugate gradient methods to remove any bad contacts between the solute and solvent atoms. The protein-membrane simulations were performed in two steps. In the first step, the lipid tails were modeled using the highly mobile membrane mimetic (HMMM) model to help better sample peptide binding and equilibrate lipid distributions around the peptide (41). In the second step, the HMMM equilibrated structure was converted to a full lipid model using CHARMM-GUI software (42, 43) The full lipid and peptide complex structure was then equilibrated for 1,000 ns at constant pressure (1 bar) and constant temperature (303K). The pressure and temperature of the system were controlled using a Parrinello–Rahman barostat (44) with a time constant of 5 ps and a Nosè–Hoover thermostat with a time constant of 1 ps, respectively (45, 46). The hydrogen atoms were constrained using the LINCS algorithm that allowed us to use a time step of 2 fs for the integrations (47). The peptide and lipid atoms were modeled using the CHARMM36 forcefield (48). All simulations were performed using GROMACS package (49) The simulation protocol described here was used in our previous studies to investigate interactions of various proteins such as Snf7, N-terminal complexin, and GP41 with the lipid membranes (50–52). All data analyses were performed using the last 500 ns of the 1,000 ns long trajectories.

### smFRET data analysis

smFRET data analysis was performed in MATLAB (Mathworks) using the SPARTAN software package (53), with additional custom-written scripts. Fluorescence traces were extracted from the single molecule movies and corrected for bleedthrough of donor fluorescence onto the acceptor channel. The corrected fluorescence traces were used to calculate the apparent FRET efficiency according to FRET = IA/(gID + IA), where IA and ID are the fluorescence emission intensities of the acceptor and donor fluorescence, respectively, and g is the empirically determined ratio of detection efficiencies on the acceptor and donor channels. smFRET trajectories were automatically identified according to several criteria: (i) donor and acceptor fluorescence trajectories displayed a single photobleaching event, which is indicative of a single FRET fluorophore pair per virion; (ii) FRET was detectable for minimally 15 frames before photobleaching; (iii) the correlation coefficient of donor and acceptor fluorescence traces was less than 0.1; and (iv) the signal-to-noise ratio of the total fluorescence, defined as the ratio of the magnitude of the photobleaching event to the variance of the background signal, was greater than 8. All smFRET trajectories that met these criteria were fit to a hidden Markov model (HMM) consisting of one or multiple states, with FRET values of 0.00 ± 0.06, 0.20 ± 0.08, 0.5 ± 0.08, and 0.96 ± 0.08 (mean ± standard deviation) depending on indicated conditions using the segmental k-means algorithm (54). All the observed FRET data points until the point of photobleaching (transition to 0 FRET) were compiled into histograms. Overlaid on the histograms are Gaussian distributions; the means and standard deviations of the Gaussian fits were constrained to reflect the results of the HMM analysis. This analysis identified the transitions between FRET states, which were used to construct transition density plots, TDPs (55).

## SUPPLEMENTAL MATERIAL

Supplemental figures mbio.01077-24-s0001.pdf Figures S1 to S17.

Table S1 mbio.01077-24-s0002.docx Fitting parameters of D614G, B.1.1.7, B.1.351, and B.1.617.2 fusion with proteoliposome in presence of PS lipid.

Table S2 mbio.01077-24-s0003.docx Fitting parameters of D614G, B.1.1.7, B.1.351, and B.1.617.2 fusion with proteoliposome in presence of varying concentrations of Lact-C2.

ASM does not own the copyrights to Supplemental Material that may be linked to, or accessed through, an article. The authors have granted ASM a non-exclusive, world-wide license to publish the Supplemental Material files. Please contact the corresponding author directly for reuse.
