# Supplementary material for: SARS-CoV-2 spike fusion peptide trans interaction with phosphatidylserine lipid triggers membrane fusion for viral entry
Source: mBio. 2024 Aug 8;15(9):e01077-24. doi: 10.1128/mbio.01077-24 (PMC11389415; doi:10.1128/mbio.01077-24)
Supplement: Supplemental figures — Figures S1 to S17. [file mbio.01077-24-s0001.pdf]

Supplemental Materials

Production of DiO-labelled lentiviral pseudovirions having SARS-CoV-2 variant spikes

Plasmids encoding different SARS-CoV-2 spikes variant (D614G/B.1.351/B.1.1.7/ B.1.617.2) and a plasmid encoding HIV-1 GagPol were transfected in HEK293T/17 cells using lipofectamine (Invitrogen), in a ratio of 5:5 of spike:gagPol (***13***). All the plasmids encoding spike variants were obtained from Stephan Pohlmann laboratory (University of Gottingen) (***6,7***). The plasmid encoding HIV-1 gagpol was a gift from Walther Mothes lab (Yale university) (***31***).

HEK293T/17 cells were maintained in DMEM (GIBCO), supplemented with 10% FBS (GIBCO), 2mM L-glutamine (GIBCO) and 100U/mL penicillin/streptomycin (GIBCO) at 37 °C, with 5% CO_2, and transfected at 70-75% confluency. 72 hours post-transfection, the supernatant was collected and filtered through a 0.45um filter to remove cell debris. Viruses were concentrated in a 10% sucrose cushion by ultra-centrifugation at 25,000xg for 2 hours. The concentrated pseudovirions were then labelled with 20uM DiO (Invitrogen) for 2 hours at room temperature in rotation. The labelled pseudovirions were purified using a 6%-30% OptiPrep gradient (Sigma-Aldrich) by centrifugation at 35,000xg for 1 hour. The labelled fractions having the virions were aliquoted and stored at -80 °C for further use.

Cloning, expression, and purification of mRFP-Lact-C2

Lactadherin-C2 (Lact-C2) tagged to mRFP was amplified from pCMV-mRFP-Lact-C2 plasmid (Addgene) (***28***). It was then cloned between NdeI and XhoI sites in the bacterial expression vector pET-28a(+). The clone was then used to transform

BL21(DE3) cells according to manufacturer's protocol (Invitrogen). Cells were grown in LB medium at 37 °C, until the O.D. reached around 0.66. Induction was done using 0.75M IPTG, at 16 °C overnight. Cells were lysed in presence of PMSF (Sigma) by sonication. After centrifugation at 18,000xg for 1 hour, the protein was first affinity purified using Ni-Sepharose beads (GE Healthcare). Lact-C2 fused mRFP has a thrombin cleavage site before the 6xHis tag at the N terminus. The His tagged domain was cleaved using thrombin (1% wt/wt). The sample was further purified using size exclusion chromatography using Superdex 200 increase 10/300 GL column (GE Healthcare). Samples was exchanged into a buffer having 20mM HEPES and 50mM NaCl for final storage. Stock solutions of the protein were stored with ~16% glycerol at -80 °C.

Cell-cell fusion assay

Two different cell lines, HEK293T/17 and Vero-TMPRSS2, were used for cell-cell fusion assay (***14***). HEK293T/17 cells were transfected with the spike plasmid of the respective variant along with GFP in a ratio of 2:2. Vero-TMPRSS2 cells were transfected with either mCherry or mRFP-Lact-C2. 24-30 hours post transfection, cells were seeded at around 1 million densities on to 0.01% poly-D-lysine (Sigma) and 4ug/mL fibronectin (Sigma) pre-treated glass bottom confocal dishes (SPL Lifesciences). HEK293T and Vero-TMPRSS2 cells were mixed in a ratio of 1:1 in DMEM supplemented with 10% FBS and Pen-Strep. The cells were allowed to attach to the confocal dishes for 24 hours, following which confocal imaging was performed **(*14*).** Images were acquired using Zeiss LSM710NLO confocal microscope under 20x objective, with a motorized XY stage for housing samples within a CO_2 enclosure and

temperature-controlled platform. For the green channel (GFP), a Multi-Line Argon laser source was used; and a diode pump solid state laser source was used was for the red channel (mCherry/mRFP). To avoid any crosstalk between the channels, the laser power and bandwidths were selected accordingly. The images acquired were processed in the Zen (Blue) software suite from ZEISS. To quantify cell-cell fusion, Mander's Coefficient was calculated using the JACoP plugin in ImageJ. Five different areas were used for calculating the mean ± SEM of the Mander's Coefficient for each case.

Virus internalization assay

HEK293T/17 cells were transfected with full-length ACE2, full-length TMPRSS2 and mRFP-Lact-C2 plasmids in a ratio of 2:2:2 (in μg). 24 hours post-transfection, the transfected cells were transferred to pre-coated confocal dishes (coated with poly-D-lysine) at a final cell density of around 1 million. The cells were allowed to attach to the dish for 24 hours, following which, the cells were infected with 100uL DiO-labelled pseudovirions. Cells were incubated with the virus at 4 °C for 10 min in order to aid greater attachment of virus to the cells. The plates were then incubated for 2 hours at 37 °C to allow the viruses to enter inside the cells. After 2 hours, the plates were washed twice with PBS, and phenol red-free DMEM was added. Confocal Data was recorded using LSM 780, Axio Observer microscope (Zeiss) using either a Plan-Apochromat 40x/1.4 Oil DIC M27 or a Plan-Apochromat 63x/1.4 Oil DIC M27 objective. mRFP-Lact-C2 was excited using 561 nm laser, and DiO-labelled virions were excited with 488 nm laser. Multiple z-stacks spanning the entire cell volume with z-planes spaced 300 nm apart were acquired.

Virus entry inhibition assay

HEK293T/17 cells were transfected with full-length ACE2 and full-length TMPRSS2 plasmids in a ratio of 2:2. The cells were seeded in confocal dishes 24 hours post-transfection. The cells were then incubated with 100nM mRFP-Lact-C2 protein diluted in DMEM at 37 °C for 45 minutes. For a control set, the cells were incubated 500nM Lysotracker Red (Invitrogen) for 10 minutes, following which the cells were washed twice with PBS. The cells were incubated with 100uL DiO-labelled virions were then added to the cells and the plates were incubated for 2 hours at 37 °C. After 2 hours, the plates were washed twice with PBS buffer, and phenol red free DMEM was added. Images were acquired in the set-up and the specifications described above at 63x magnification by a 1.4 NA oil-based objective. Analysis was performed in Zen Blue and ImageJ. The number of virions were counted using automatic counting in ImageJ by defining a threshold.

Liposome preparation

Liposomes with phosphatidylserine (PS) were prepared in a 4:4:0.5:0.1:2 ratio of 1,2, dioleoyl-sn-glycero-3-phosphocholine (DOPC; Avanti Polar Lipids), 1-palmitoyl-2-oleoyl-glycero-3-phosphocholine (POPC; Avanti Polar Lipids), phosphatidylserine (PS; Avanti Polar Lipids), Ni-NTA DGS lipid (Avanti Polar Lipids) and cholesterol (Avanti Polar Lipids) (***13, 30, 31***). Briefly, the lipids were mixed in chloroform, which was evaporated under a stream of argon gas. The dried lipid film was resuspended in HNE buffer (5mM HEPES, 145mM NaCl; pH 7.5), which was followed by five freeze-thaw cycles. Liposomes of around 100nm were obtained by extruding the homogenized aqueous lipid suspension through a 100nm polycarbonate membrane

filter. The freshly prepared liposomes were coated with histidine-tagged hACE2, NRP1-b1 and hTMPRSS2 in a ratio of 1:1:1 at 37 °C for at least 1 hour in rotation ***(13)***. Liposomes without phosphatidylserine (PS) were prepared in a 4:4:0.1:2 ratio of 1,2, dioleoyl-sn-glycero-3-phosphocholine (DOPC; Avanti Polar Lipids), 1-palmitoyl-2-oleoyl-glycero-3-phosphocholine (POPC; Avanti Polar Lipids), Ni-NTA DGS lipid (Avanti Polar Lipids) and cholesterol (Avanti Polar Lipids).

Liposomes for smFRET imaging were prepared by the protocol described above. Liposomes having NBD-PS were prepared in a 4:4:0.000625:0.1:2 ratio of 1,2,dioleoyl-sn-glycero-3-phosphocholine (DOPC; Avanti Polar Lipids), 1-palmitoyl-2-oleoyl-glycero-3-phosphocholine (POPC; Avanti Polar Lipids), 1,2-dipalmitoyl-*sn*-glycero-3-phosphotidylserine-N-(7-nitro-2-1,3-benzoxadiazol-4-yl) (NBD PS; Avanti Polar Lipids, cat# 810194)., Ni-NTA DGS lipid (Avanti Polar Lipids), and cholesterol (Avanti Polar Lipids). Liposomes having NBD-PE were prepared in a 4:4:0.000625:0.1:2 ratio of 1,2,dioleoyl-sn-glycero-3-phosphocholine (DOPC; Avanti Polar Lipids), 1-palmitoyl-2-oleoyl-glycero-3-phosphocholine (POPC; Avanti Polar Lipids), 1,2-dipalmitoyl-*sn*-glycero-3-phosphotidylethanolamine-N-(7-nitro-2-1,3-benzoxadiazol-4-yl) (NBD PE; Avanti Polar Lipids, cat# 810155), Ni-NTA DGS (Avanti Polar Lipids), and cholesterol (Avanti Polar Lipids). The liposomes were coated with recombinant hACE2, NRP1-b1 and hTMPRSS2 with the following molar concentration of lipids and proteins: 1,2, dioleoyl-sn-glycero-3-phosphocholine (DOPC; Avanti Polar Lipids): 31.8 mM, 1-palmitoyl-2-oleoyl-glycero-3-phosphocholine (POPC; Avanti Polar Lipids): 32.9 mM, Phosphatidylserine (PS; Avanti Polar Lipids): 6 mM, Ni-NTA DGS lipid (Avanti Polar Lipids): 5 mM, Cholesterol (Avanti Polar Lipids):

25 mM, Recombinant ACE2: 1.8 μM, Recombinant NRP1: 1.8 μM, Recombinant TMPRSS2: 1.8 μM.

Virus-liposome lipid mixing assay

The virus liposome lipid mixing assay has been described extensively in our previous study ***(13).*** Briefly, DiO-labelled pseuvirions with either WT spike or D614G spike were combined with proteo-liposomes (either with or without PS) coated with hACE2, NRP1-b1 and hTMPRSS2. Buffer solution was added in a stop-flow manner to adjust desired pH conditions and DiO fluorescence was followed in a time-based manner. DiO was excited at 488 nm and fluorescence was detected at 515 nm at every second's intervals for 20 minutes in a QuantaMaster fluorescence spectrophotometer 8450 (Horiba). All the fusion experiments were performed at 37C by rapid Peltier temperature-controlled sample holder. Data was acquired with the FelixGx software provided by the manufacturer. All the data was analyzed and plotted using MATLAB. For Lact-C2 titration, different concentrations of Lact-C2, ranging from 0pM-25nM were used. Liposomes having Ni-NTA DGS were incubated with the designated concentration of Lact-C2 (possessing His-tag) for 60 mins in rotation. The liposomes were also coated with ACE2, NRP1 and TMPRSS2 along with Lact-C2. The fusion reaction was carried out in the same manner described above.

Virus fusogenicity assay

The fusogenicity of the pseudotyped virions was tested by a β-lactamase (BlaM)-based enzymatic assay (LiveBlazer FRET B/G kit, Invitrogen) (***29, 31***). The pseudoviruses were formed as described above, but with an additional plasmid

encoding BlaM, fused to the HIV-1 Vpr protein. Viruses were collected and concentrated as described above and resuspended in phenol red-free DMEM (GIBCO), supplemented with 10% FBS (GIBCO), 2mM L-glutamine (GIBCO) and 100U/mL penicillin/streptomycin (GIBCO). Vero-TMPRSS2 cells were seeded in 96-well plates and incubated with either Lact-C2 or exogenous phosphatidylserine (PS). Lact-C2 diluted in DMEM was used at a final concentration of 100nM, and PS in chloroform diluted in MilliQ water was used at a final concentration of 2.5uM. The virions were then used for infecting HEK293T/17 cells. Spinoculation was done at 3700 rpm at 4 °C. Unbound viruses were removed by washing with HBSS (GIBCO) and resuspending the cells in fresh phenol red-free DMEM. The plate was then incubated at 37 °C for 90 minutes to permit viral entry. Cells were then loaded with the substrate, CCF4-AM fluorophore, in presence of probenecid at a final concentration of 2.5mM. The plate was incubated overnight at 11 °C. The cleavage of CCF4-AM by BlaM was detected using a plate reader (Biotek). The fusogenicity of the virus was calculated as a ratio of blue to green emission.

Incorporation of non-canonical amino acid in the SARS-CoV-2 spike (S*)

A single non-canonical amino acid (ncAA), TCO*, was incorporated into the S2 domain of SARS CoV-2 spike through amber stop codon suppression technology, as described previously ***(30, 31)***. TAG codon was introduced at the position 836 of S2 near the fusion peptide proximal residue (FPPR). Translation to incorporate the ncAA was allowed to proceed through the UAG codons on the mRNA in the presence of an orthogonal tRNA ($tRNA^{Pyl}$), which recognizes the UAG codon. A corresponding aminoacyl-tRNA synthetase ($NESPylRS^{AF}$) was used which aminoacylates the

suppressor tRNA with TCO*, facilitating its incorporation at the 836th position into S, forming S*. The efficiency of amber suppression is limited due to competition of the eukaryotic release factor 1 (eRF1) with tRNA^{Pyl}. Expression of the dominant negative eRF1 E55D mutant increased amber suppression efficiency ***(54)***.

HEK293T cells were transfected with plasmids encoding the spike, TAG-mutated S*, NESPylRS^{AF}/tRNA^{Pyl}, eRF1 E55D, and HIV-1 Gag-Pol in a ratio of 2.5:2.5:2.5:1.5:5. The growth medium was supplemented with 500uM TCO* ncAA (SiChem). At 72 hours post-transfection the virus was harvested by using a 0.45um syringe filter. The virions were concentrated in a 10% sucrose cushion by centrifugation at 25,000xg at 4 C. The concentrated virions were labelled with DSPE-Biotin for 30 minutes in rotation, following which 500uM Cy5 was added. Cy5-labelled virions were purified in a 6%-30% Opti-Prep gradient by centrifugation at 35,000xg for 1 hour. The fractions having the labelled virions were collected and used for smFRET imaging (**Figure S12**).

For the cis-interaction assay, pseudovirions with S*-Cy5, and labelled with DSPE-Biotin, were incubated with a final concentration of 0.6nM NBD-PS for one hour in rotation, so that NBD-PS is incorporated in the membrane of the virions. Labelled virions were then purified using 6-30% Opti-Prep, as described above.

smFRET Imaging of SARS CoV-2 virions and PS lipid interaction

smFRET imaging was done using a home-built prism-based TIRF microscope. Quartz slides passivated with PEG (polyethylene glycol) were used for imaging. Slides were coated with streptavidin to immobilize DSPE-Biotin tagged virions. Liposomes were flowed once the virions were immobilized on the slide. Unbound liposomes were

removed by washing with 100uL of triggering solution. The slides were mounted on an XY-piezo stage (Applied Scientific Instruments). An evanescent field was created by total internal reflection using a 488-nm solid state laser (Coherent), at a power of 150mW. Fluorescence emission was collected with a 60x water-immersion objective. Donor and acceptor emissions were separated using dichroic filter and the emissions were collected using synchronized sCMOS cameras (Hamamatsu). Images were acquired at 5 frames/millisecond using custom-built MicroManager software. The imaging cocktail contained 50mM Tris (pH 8.0) and 50mM NaCl, along with triplet state quenchers (1mM trolox, 1mM cyclooctatetraene, 1 mM nitrobenzyl alcohol) and beta-mercaptoethanol **(*34–35*).** The cocktail also included an enzymatic system for removal of molecular oxygen, which included 2 mM protocatechuic acid (PCA) and 8 nM protocatechuate 3,4-deoxygenase (PCD) ***(34, 35)***. The buffers for different pHs were made using a combination of citric acid and disodium hydrogen phosphate, and the triplet state quenchers and oxygen scavengers were added to them.

For the cis-interaction assay, after immobilizing viruses on the PEGylated slide, triggering solution (pH 4.6/pH 4.6 & 500uM Ca^{2+}/pH 7/pH 7 & 500uM Ca^{2+}) was flowed. Imaging was performed using the same conditions as described above. The conformation reversibility experiment involving EDTA was performed by incubating the virions with pH 4.6 & 500uM Ca^{2+} for 15 mins/ 30 mins (as indicated) and then flowing 50mM EDTA in order to chelate all the Ca^{2+} present in the surrounding. All the pH and EDTA solutions contained the components of the imaging cocktail in order to prevent quenching of the fluorophores.

Viral membrane phosphatidylserine-Lact-C2 colocalization assay

Virus-like particles (VLPs) having spike, envelope, membrane and nucleocapsid proteins (S-E-M-N) proteins were prepared by transfecting all the plasmids in HEK293T/17 cells in 5:5:5:5 ratio ***(13).*** VLPs with B.1.617.2 and D614G spike were produced. 72 hours post transfection, the VLPs were collected and concentrated using the steps described above. The virions were labelled with DiO, and DSPE-Biotin, and the labelled virions were separated from free dye using OptiPrep and density gradient centrifugation as mentioned before. The labelled VLPs were flowed on pegylated quartz slide and incubated for 30 mins, following with recombinant Lact-C2 labelled with mRFP was flowed in the same channel (**Figure S15**). The VLPs were incubated with Lact-C2 for 30 mins in order to allow the Lact-C2 to bind to the viral membrane phosphatidylserine, if present. The channel was the washed twice with 100uL T50 buffer to remove unbound or excess Lact-C2. The slide was mounted on an XY-piezo stage. VLPs were imaged using a 488-nm blue laser, and a green laser was used for imaging Lact-C2. Images acquired were then analyzed and merged using ImageJ ***(55)***.

MD Simulations

Initial coordinates of the fusion peptide (S2) were taken from the protein data bank (PDB Id: 7KRQ). A single S2 fusion peptide proximal residues (816-855 aa) was placed on top of a lipid bilayer consisting of DOPC, POPC, PS lipids and cholesterols. The membrane-protein complex was then solvated using TIP3P water box ***(36)***. Appropriate number of Ca^{2+} and Cl^{-} were added to maintain charge neutrality. The built structure was then energy minimized using the steepest descent and conjugate

gradient methods to remove any bad contacts between the solute and solvent atoms. The protein-membrane simulations were performed in two steps. In the first step, the lipid tails were modeled using the Highly Mobile Membrane Mimetic (HMMM) model to help better sample peptide binding and equilibrate lipid distributions around the peptide ***(37)***. In the second step, the HMMM equilibrated structure was converted to full lipid model using CHARMM-GUI software ***(38, 39)*** The full lipid and peptide complex structure was then equilibrated for 1000 ns at constant pressure (1 bar) and constant temperature (303K). The pressure and temperature of the system were controlled using a Parrinello–Rahman barostat ***(40)*** with a time constant of 5 ps and a Nosè–Hoover thermostat with a time constant of 1 ps, respectively ***(41, 42)*** The Hydrogen atoms were constrained using the LINCS algorithm that allowed us to use a time step of 2 fs for the integrations ***(43)***. The peptide and lipid atoms were modeled using the CHARMM36 forcefield ***(44)***. All simulations were performed using GROMACS package ***(45)*** The simulation protocol described here was used in our previous studies to investigate interactions of various proteins such as Snf7, N-terminal Complexin and GP41 with the lipid membranes ***(46-48)***. All data analysis were performed using the last 500 ns of the 1000 ns long trajectories.

smFRET data analysis

smFRET data analysis was perform in MATLAB (Mathworks) using the SPARTAN software package ***(49)***, with additional custom-written scripts. Fluorescence traces were extracted from the single molecule movies and corrected for bleedthrough of donor fluorescence onto the acceptor channel. The corrected fluorescence traces were used to calculate the apparent FRET efficiency according to FRET= IA/(gID+IA),

where IA and ID are the fluorescence emission intensities of the acceptor and donor fluorescence, respectively, and g is the empirically determined ratio of detection efficiencies on the acceptor and donor channels. smFRET trajectories were automatically identified according to several criteria: (1) donor and acceptor fluorescence trajectories displayed a single photobleaching event, which is indicative of a single FRET fluorophore pair per virion; (2) FRET was detectable for minimally 15 frames before photobleaching; (3) the correlation coefficient of donor and acceptor fluorescence traces was less than 0.1; (4) the signal-to-noise ratio of the total fluorescence, defined as the ratio of the magnitude of the photobleaching event to the variance of the back- ground signal, was greater than 8. All smFRET trajectories that met these criteria were fit to a hidden Markov model (HMM) consisting of one or multiple states, with FRET values of 0.00 ± 0.06, 0.20 ± 0.08, 0.5 ± 0.08, and 0.96 ± 0.08 (mean ± standard deviation) depending on indicated conditions using the segmental k-means algorithm ***(50)***. All the observed FRET data points until the point of photobleaching (transition to 0 FRET) were compiled into histograms. Overlaid on the histograms are Guassian distributions; the means and standard deviations of the Gaussian fits were constrained to reflect the results of the HMM analysis. This analysis identified the transitions between FRET states, which were used to construct transition density plots, TDPs ***(51)***.

Supplementary figures

Figure S1

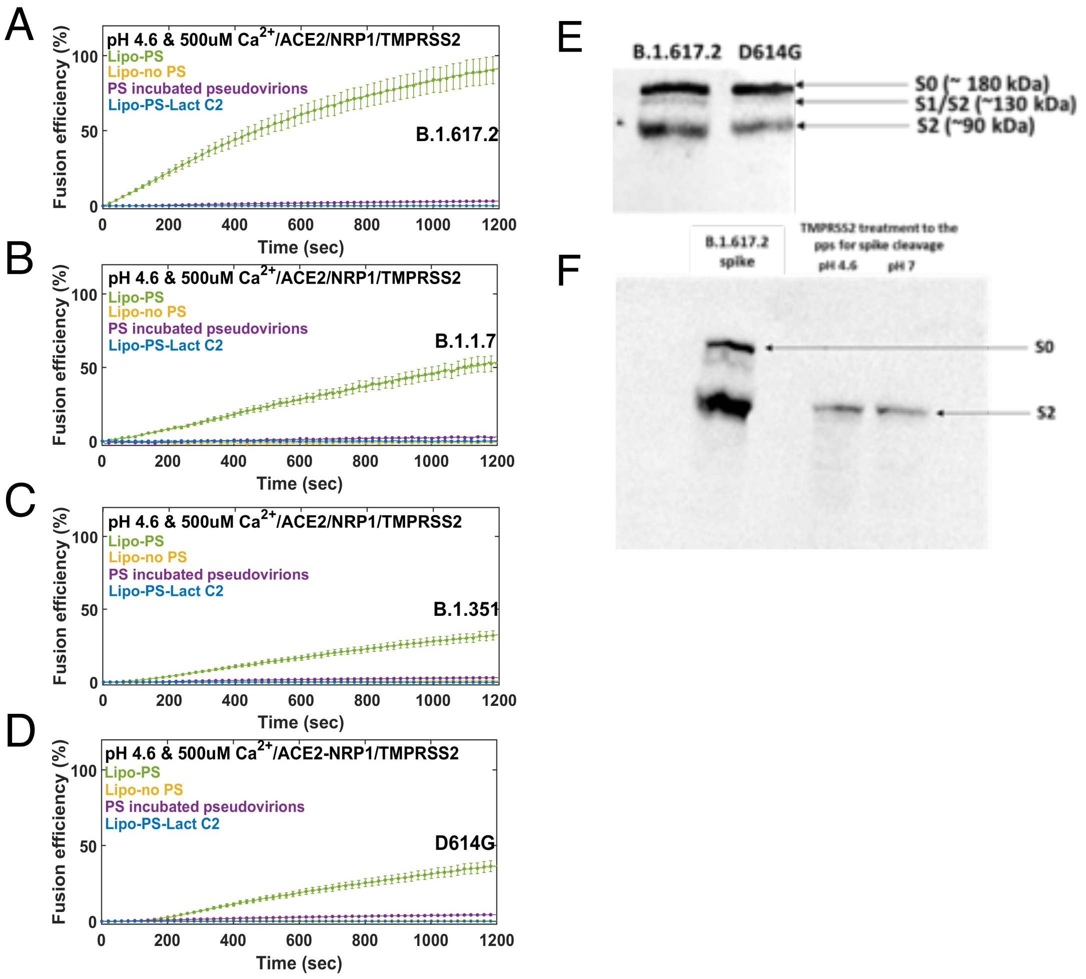

Figure S1. PS lipid is indispensable for SARS-CoV-2 spike mediated virus-liposome membrane fusion. PS in target membrane stimulates fusion and PS in viral membrane abrogates fusion. Fusion efficiencies based on fluorescence dequenching assay were monitored for 20 minutes for **(A)** B.1.617.2 spike pseudovirions, **(B)** B.1.1.7 spike pseudovirions, **(C)** B.1.351 spike pseudovirions, and **(D)** D614G spike pseudovirions. For all the spike variant, robust fusion was observed when PS is present in the liposome. In absence of PS, no fusion is observed. No fusion efficiency was observed for incubation of PS with the viral membrane or

sequestering PS with Lact-C2 recombinant protein. Error bars represent data in triplicates. **(E)** Western blot data showing the expression of spike on the pseudoparticles. Both the B.1.617.2 and D614G spike is found to be cleaved at the S1/S2 region in the respective pseudovirions particles. **(F)** Western blot data showing the functional activity of TMPRSS2 in spike cleavage at low pH and neutral pH conditions.

Figure S2

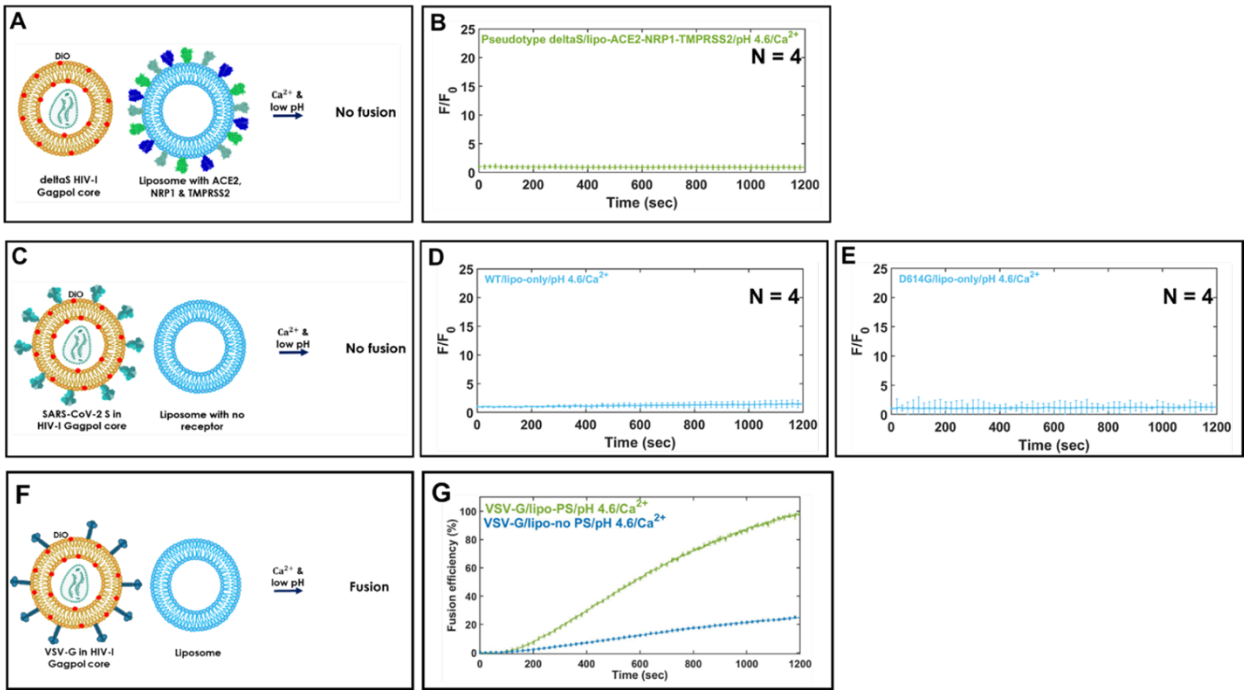

Figure S2: Control experiments shows that virus and liposome fusion is mediated by SARS-CoV-2 spike and receptor. (A) Schematic of fusogenicity assay for empty pseudoparticles without spike, with liposomes coated with ACE2, NRP1 and TMPRSS2. **(B)** Fluorescence dequenching data for the empty pseudoparticles and receptor coated liposomes. **(C)** Assay figure depicting fusogenicity assay for pseudoparticles with spike, and liposomes not coated with any receptor proteins. **(D, E)** Fluorescence dequenching data for the fusion assay with wild type spike **(D)** and D614G spike **(E)** with pseudoparticles and uncoated liposomes. **(F,G)** Schematic assay figure for positive control experiment for pseudoparticles presenting VSV-G **(F). (G)** The fluorescence dequenching reaction was performed in the presence of PS-positive liposomes (green curve) and PS-negative liposomes (blue curve).

Figure S3

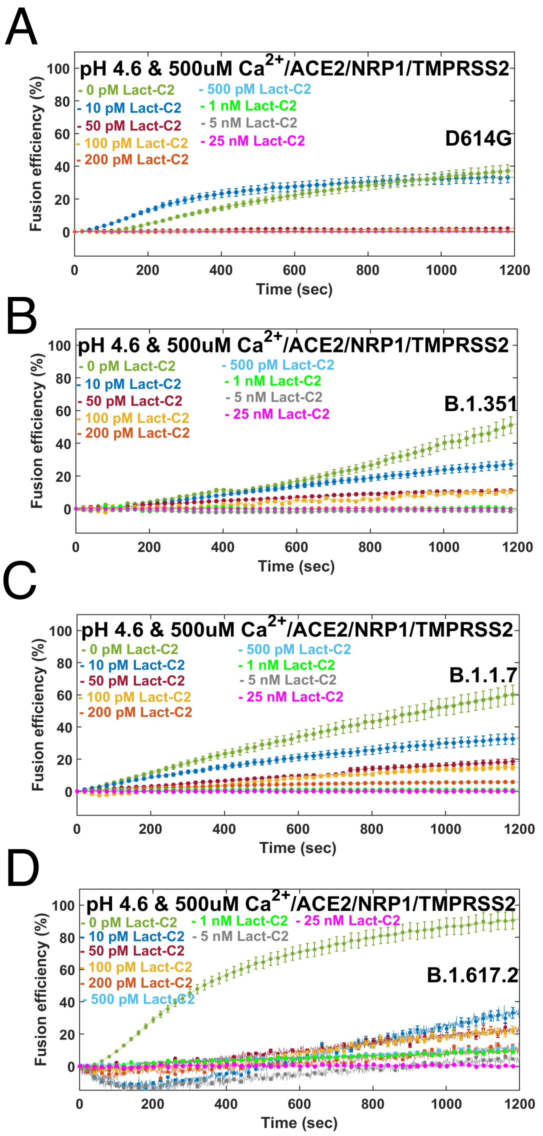

Figure S3. Sequestering PS lipid with Lact-C2 protein inhibits SARS-CoV-2 spike mediated virus-liposome membrane fusion. Steady state membrane fusion assay based on DIO fluorescence dequenching was performed under varying concentrations of Lact-C2, ranging from 10pM to 25nM. Fusion efficiencies for **(A)** D614G spike pseudovirions, **(B)** B.1.351 spike pseudovirions, **(C)** B.1.1.7 spike pseudovirions, and **(D)** B.1.617.2 spike pseudovirions in presence of varying concentration of Lact-C2. Each panel is a combination of fusion efficiencies for different Lact-C2 concentrations as indicated. Error bars represent data in triplicates.

Figure S4

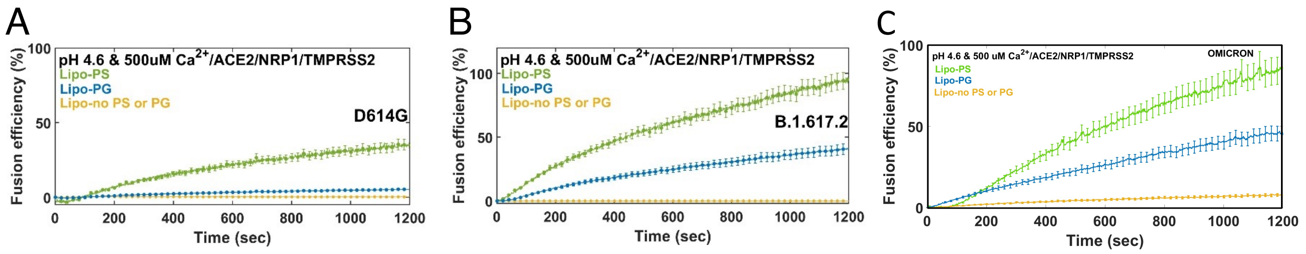

Figure S4. PS lipid is more efficient compared to PG lipid in SARS CoV-2 spike mediated virus-liposome lipid mixing. (A) No detectable fusion is observed in presence of PG lipid for D614G spike **(B,C)** lipid mixing efficiency for B.1.617.2 spike and omicron BA.2 spike has been found to be 50% in presence of PG lipid compared to the PS lipid. This indicates that PS lipid is more efficient compared to PG lipid for promoting spike mediated fusion.

Figure S5

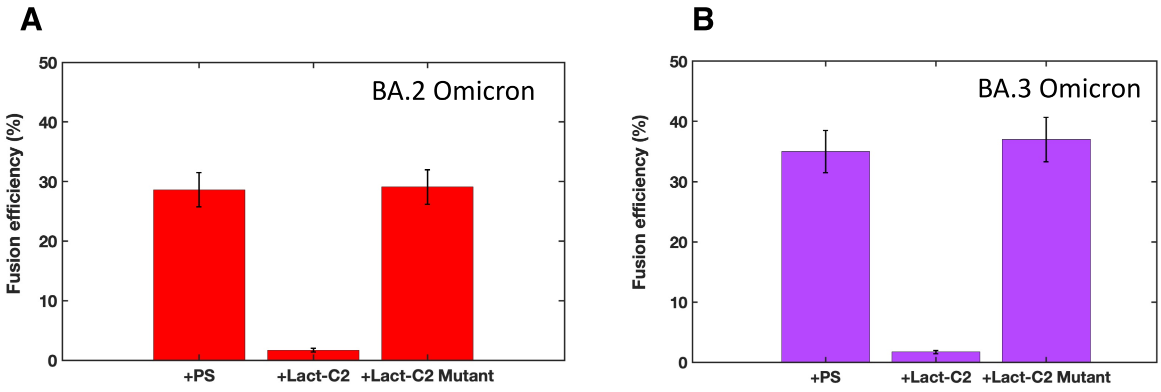

Figure S5. Lact-C2 inhibits spike mediated fusion via interaction with PS lipid.

(A, B) Mutant Lact-C2 is unable to inhibit the membrane fusion shown for BA.2 **(A)** and BA.3 **(B)** omicron spike strains.

Figure S6

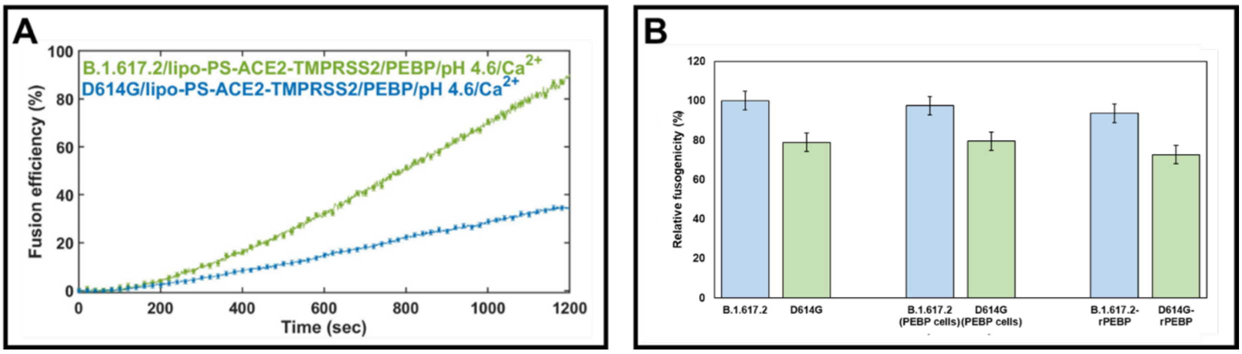

Figure S6: (A) Control lipid mixing data for pseuodparticle fusion to liposomes in presence of PE lipid binding protein (PEBP1). The lipid mixing reaction was performed for pseudoparticles with B.1.617.2 spike (green curve) and pseudoparticles with D614G spike (blue curve) in presence of PEBP1. **(B)** Beta-lactamase fusogenicity assay for Delta and D614G pseudovirions, with Vero-TMPRSS2 as the target cells. PEBP cells refer to Vero-TMPRSS2 cells expressing PEBP. Delta/D614G-rPEBP refers to cells incubated with recombinant PEBP protein, and then infected with pseudovirions. The B.1.617.2 / D614G spike fusogenicity was retained in presence of PE lipid bind proteins.

Figure S7

A

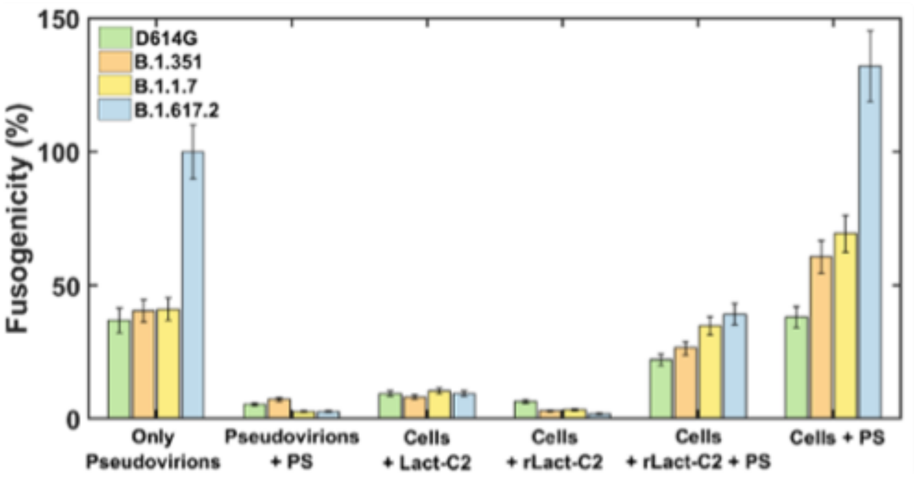

B

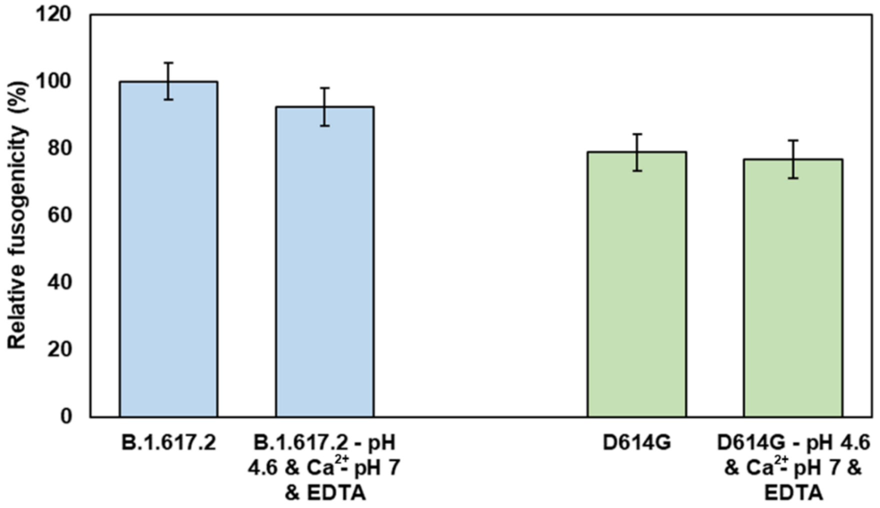

Figure S7. SARS CoV-2 fusion activity is PS lipid dependent.

(A) BlaM Enzymatic based fusiogenicity assay for SARS CoV-2 spike psedovirions were performed for different variants (D614G/ B.1.351/B.1.1.7/ B.1.617.2) to quantify the virus fusogenicity difference as per indicated conditions **(Methods)**. The data shows that incubating PS lipid with virions lowers the fusion, whereas incubating PS lipid with target cell enhances the fusion ability. Presence of exogenous expression of Lact-C2 or recombinant Lact-C2 protein (rLact-C2) inhibits the spike fusion activity. The fusogenicity data for each different conditions were plotted for four spike

pseudotype variants (D614G / Alpha / Beta / Delta variants). **(B)** Beta-lactamase fusogenicity assay for spike pseudoparticles (B.1.617.2 and D614G variants) after exposure to low pH and Ca^{2+} for 5 minutes, followed returning to neutral pH and EGTA, versus pseudoparticles not subjected to any such pre-triggering treatment.

Figure S8

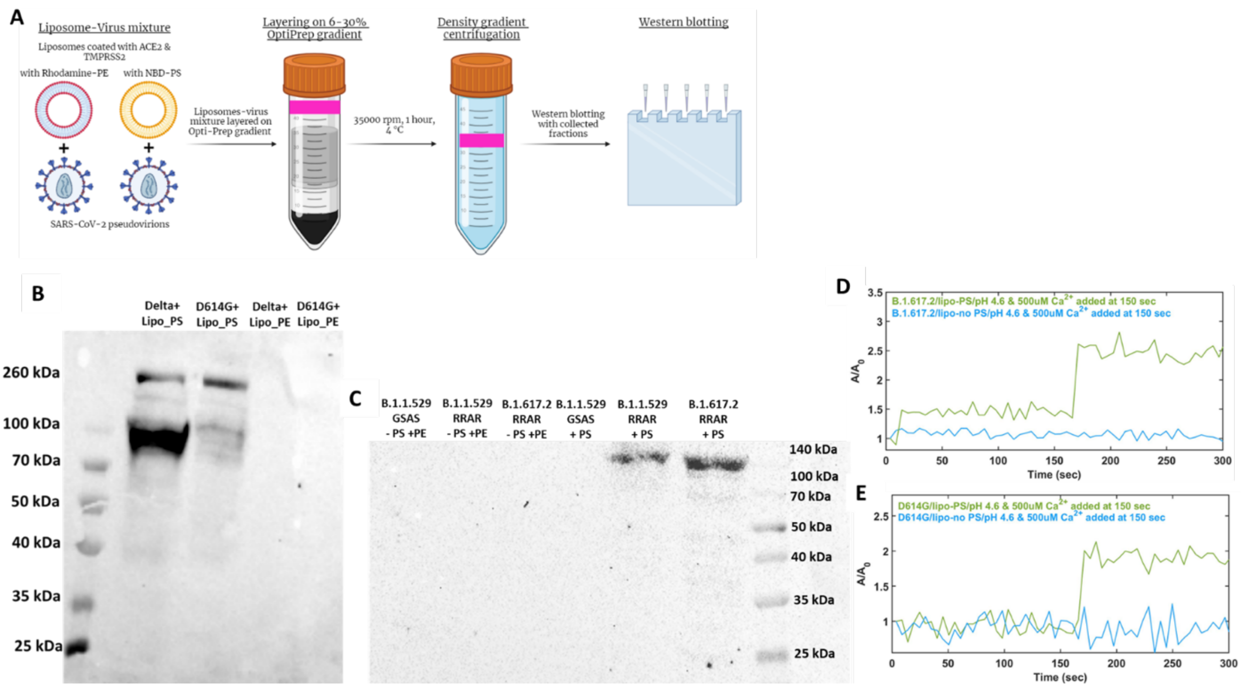

<u>Figure S8:</u> **(A)** Schematic assay depicting lipid floatation assay **(methods)**. **(B)** Western blot data for lipid floatation assay for showing pseudovirons spike binding to liposomes with PS (Lipo_PS), and liposomes without PS (Lipo_PE). **(C)** Western blot data for lipid floatation assay for showing ectodomain spike binding to liposomes with PS (Lipo_PS), and liposomes without PS (Lipo_PE). **(D)** Fluorescence anisotropy data for B.1.617.2 pseudovirions in presence of liposomes with and without PS. **(E)** Fluorescence anisotropy data for D614G pseudovirions in presence of liposomes with and without PS.

Figure S9

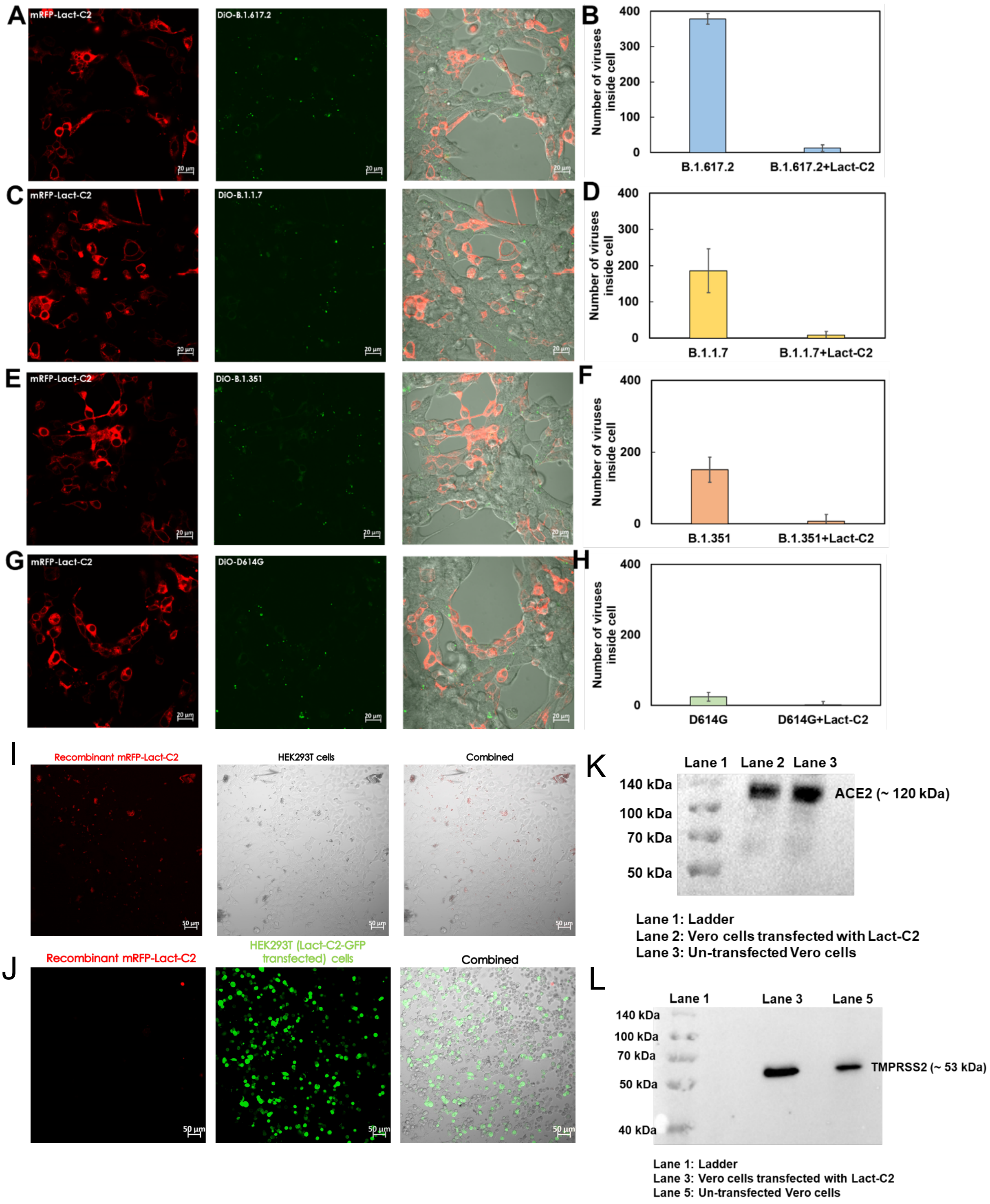

Figure S9. Expression of Lact-C2 inhibits SARS CoV-2 pseudovirus (Delta, Alpha, Beta, D614G) internalization into host cell. The assay was performed for

assessing if target cells (HEK293T cells expressing full-length ACE2 and TMPRSS2) can be infected with the virions if Lact-C2 is being expressed in the cells. The assay was performed for pseudovirions tagged with DiO, with 4 different spike strains: **(A, B)** B.1.617.2 spike, **(C,D)** B.1.1.7 spike, **(E,F)** B.1.351 spike, and **(G,H)** D614G spike. Cells in which Lact-C2 is present appear red, as Lact-C2 is tagged with mRFP. Pseudovirions are green in colour. Virion number was quantified using ImageJ. Replicates of 5 images were used for each set, and for each spike mutant. Scale bars are mentioned at the bottom right panel of each confocal image. Error bar is in standard error of mean. **(I)** Confocal images for HEK293T cells incubated with recombinant mRFP-Lact-C2 protein to assess the external PS levels in the cell surface. **(J)** Confocal images for HEK293T cells transfected with Lact-C2 expressing GFP, incubated with recombinant mRFP-Lact-C2 protein to assess the external PS levels in the cells while transfected with Lact-C2. **(K,L)** Western blot analysis for ACE2 **(K)** and TMPRSS2 levels **(L)** in Vero-TMPRSS2 cell line with and without Lact-C2 transfection.

Figure S10

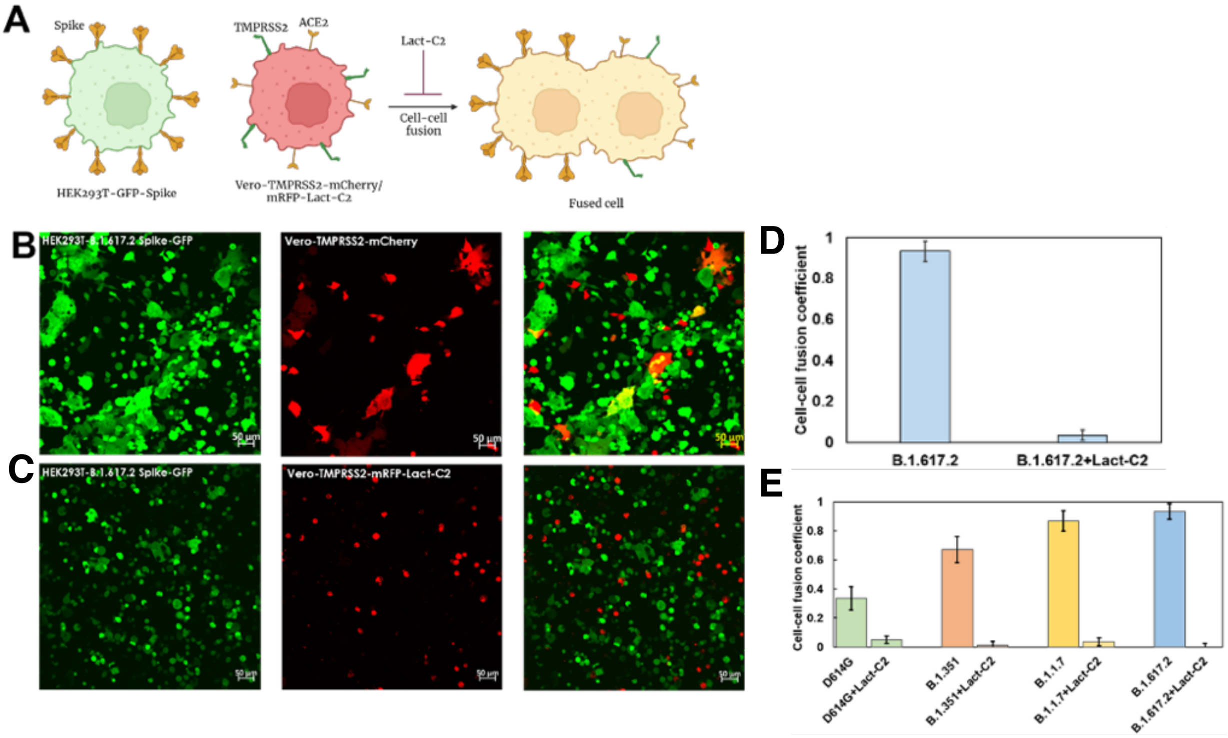

Figure S10. Phosphatidylserine (PS) is required for SARS-CoV-2 spike mediated cell-cell fusion, Sequestering PS with Lact-C2 restricts syncytia formation. (A) Schematic representation of the SARS CoV-2 spike mediated cell-cell fusion assay. HEK293T cells expressing SARS-CoV-2 spike variants (D614G / Alpha / Beta / Delta variants), and GFP (green cell) and Vero-TMPRSS2 cells expressing mCherry (red cell) were incubated and cultured together. Fusion between the HEK293 and Vero cells form syncytia and marked by the appearance of large yellow cells. **(B)** Cell-Cell fusion with the B.1.617.2 spike variants, being expressed by HEK293T cells, along with GFP. The spike expressed 293T cells were able to fuse with the target Vero-TMPRSS2 cells and forms syncytia. **(C)** Expression of Lact-C2 fused with mRFP in the Vero cell inhibits fusion and syncytia formation. **(D)** Fusion was quantified via colocalization of the green and red cells by using mander's coefficient. Data were

plotted from 3 replicates of independent set of experiments (N=3). **(E)** Cell-cell fusion coefficient was plotted in normal conditions and compared with the presence of Lact-C2. for the other spikes variants: B.1.1.7, B.1.351 and D614G spikes. Quantification was done for all the strains, and plotted as a comparative bar plot. Presence of Lact-C2 abrogates the Cell-Cell fusion for all these spike variants.

Figure S11

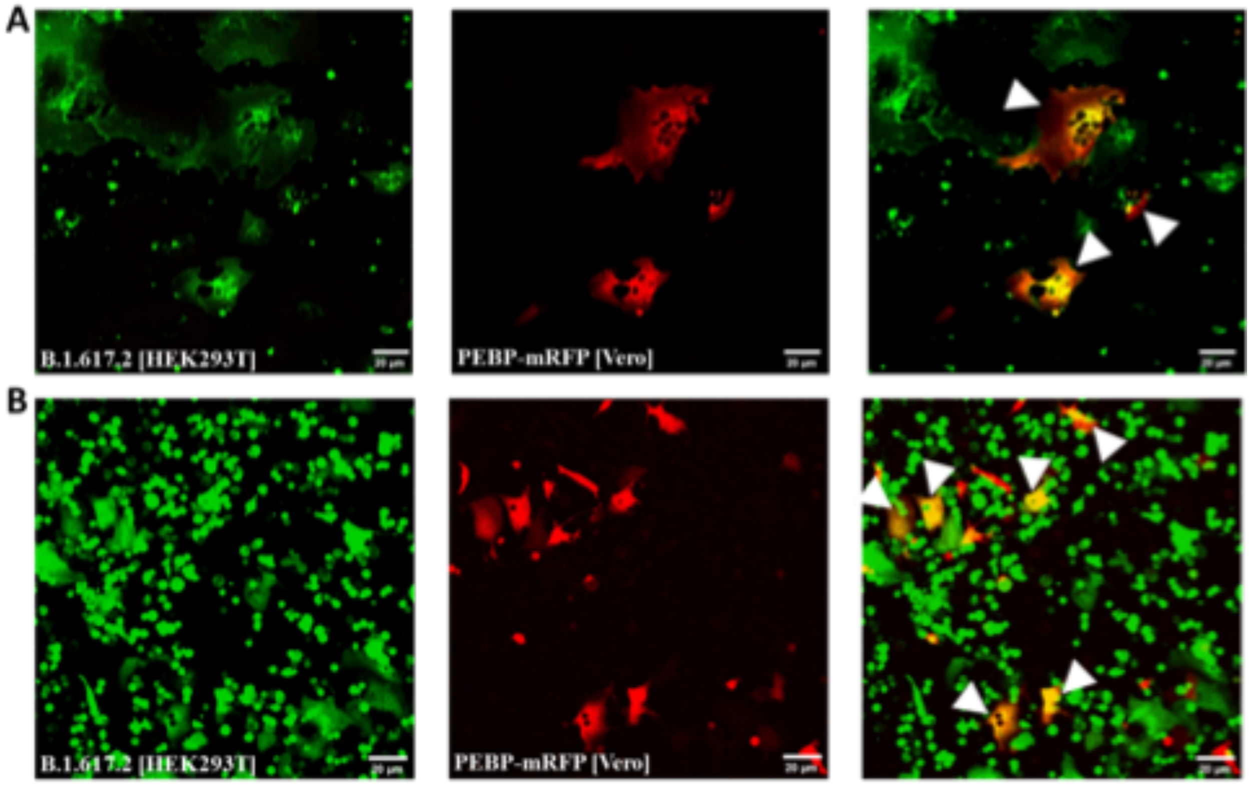

Figure S11: (A & B) The left panel shows HEK293T cells expressing B.1.617.2 spike with GFP. The middle panel shows Vero cells expressing PEBP with mRFP. The right panel shows merged channel image for the green HEK293T cells and red Vero cells. The yellow cells (highlighted with white arrowheads) are indicative of cell-cell fusion, showing that PEBP expression does not hinder fusion of spike to the host cell.

Figure S12

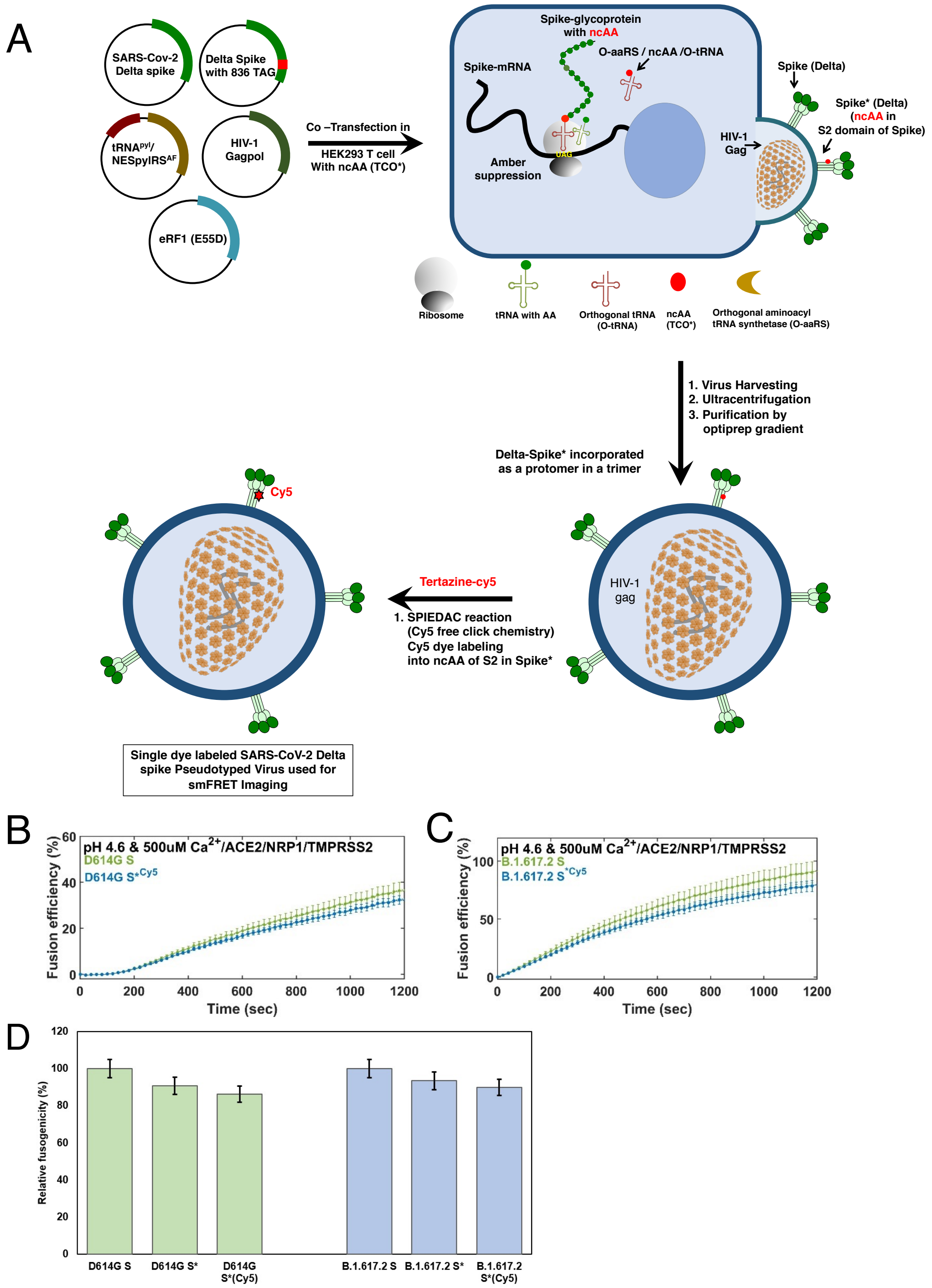

Figure S12. Schematics outlining the steps involved for pseudotyped SARS CoV-2S B.1.617.2 or D614G spike virions generation with fluorescent labelled spike trimer using genetic expansion code techniques. (A) Schematic outlining the steps involved in production of fluorescently labeled (Tetrazine Cy-5) pseudotyped virions

with SARS-CoV-2 Delta or D614G spike, for smFRET measurements, using orthogonal translation system and amber stop codon suppression. See **METHODS** for details. (**B**) Comparison of fusion activity with pseudovirions containing 100% D614G spike or 100% S*-Cy5 (D614G). (**C**) Comparison of fusion activity with pseudovirions containing 100% B.1.617.2 or 100% S*-Cy5 (B.1.617.2). (**D**) Relative fusogenicity with pseudovirions containing either 100% D614G Spike, 100% S*, or 100% S*-Cy5 for D614G **(left green)** and 100% B.1.617.2 spike, 100% S*, or 100% S*-Cy5 for B.1.617.2 **(right blue)** See **METHODS** for details. The S* and S*-Cy5 is 90% and 85% fusogenic relative to D614G-S respectively and B.1.617.2-S* and B.1.617.2-S*-Cy5 is 95% and 90% fusogenic relative to B.1.617.2 spike respectively. (N = 4 independent experiments; error bar is in standard error of mean).

Figure S13

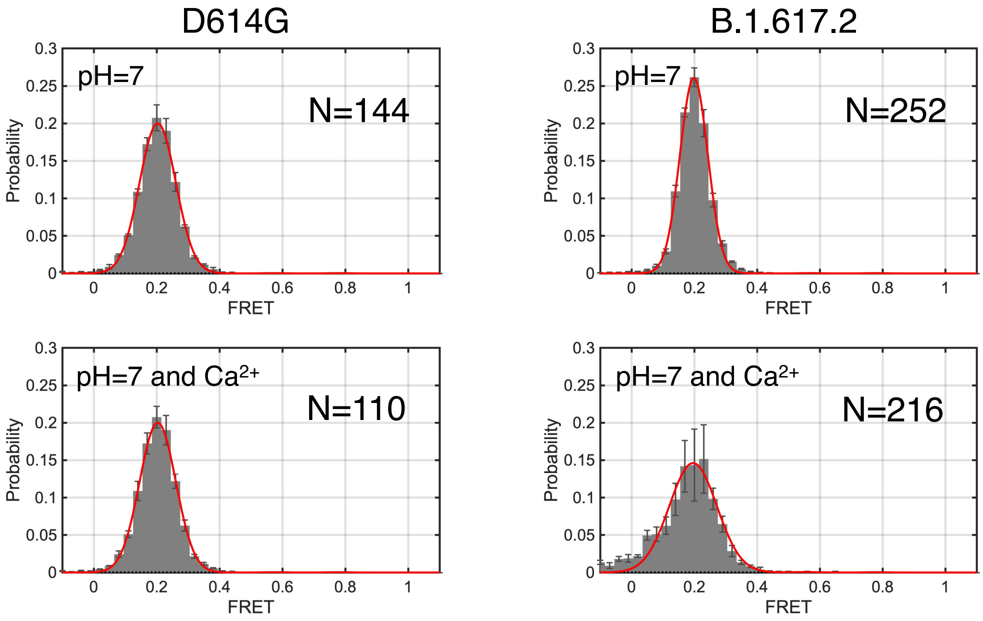

Figure S13. No high FRET state was observed between SARS CoV-2 spike fusion peptide and PS lipid at neutral pH condition and in presence of Ca^{2+}. (A). smFRET histogram for imaging D614G-Cy5 virions and NBD-PS liposomes at pH 7 and pH 7 & 500uM Ca^{2+}. **(B).** smFRET histogram for imaging B.1.617.2-Cy5 virions and NBD-PS liposomes at pH 7 and pH 7 & 500uM Ca^{2+}.

Figure S14

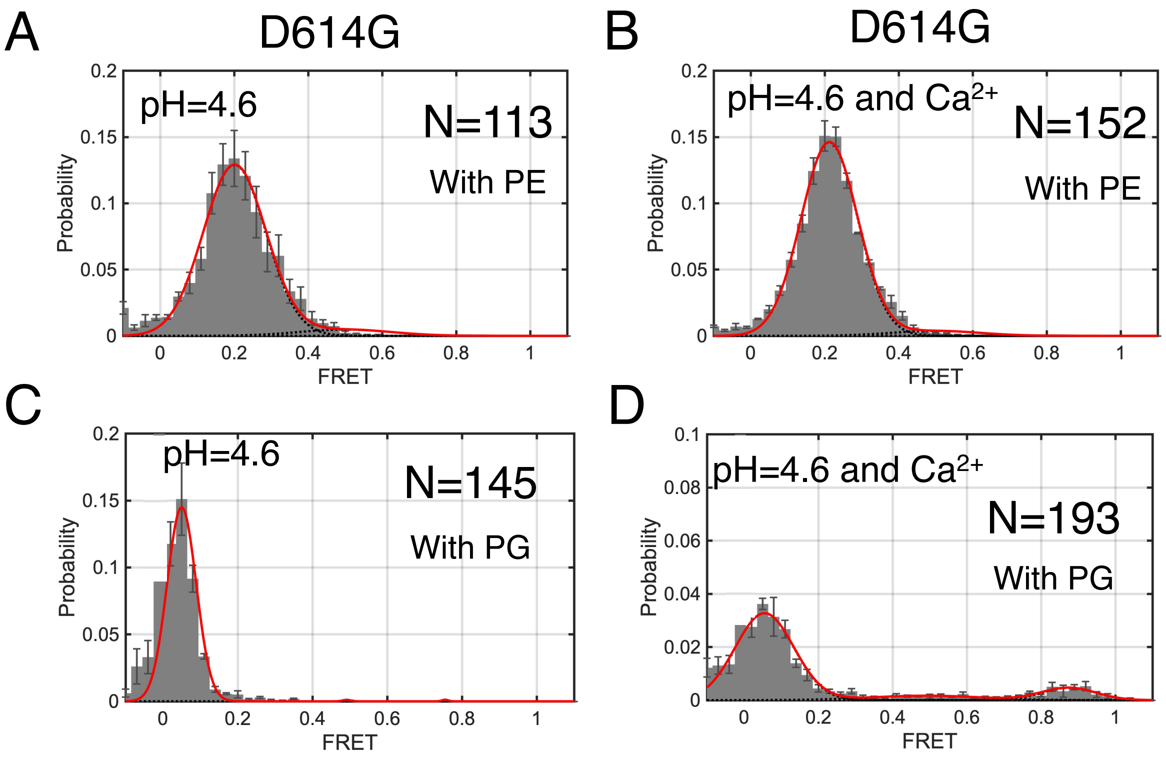

Figure S14. smFRET histogram between SARS CoV-2 spike fusion peptide and PE Lipid and PG lipid at low pH and Ca^{2+} fusion condition. (A,B) Histogram for smFRET imaging with D614G-Cy5 virions and NBD-PE liposomes at **(A)** pH 4.6 and **(B)** pH 4.6 & 500uM Ca^{2+}, when PS is not present on the liposomes. NBD-PE was used as donor dye for the assay. **(C,D)** Histogram for smFRET imaging with D614G-Cy5 virions and NBD-PG liposomes at **(C)** pH 4.6 and **(D)** pH 4.6 & 500uM Ca^{2+}, when no PS lipid is present on the liposomes. NBD-PG was used as donor dye for the assay.

Figure S15

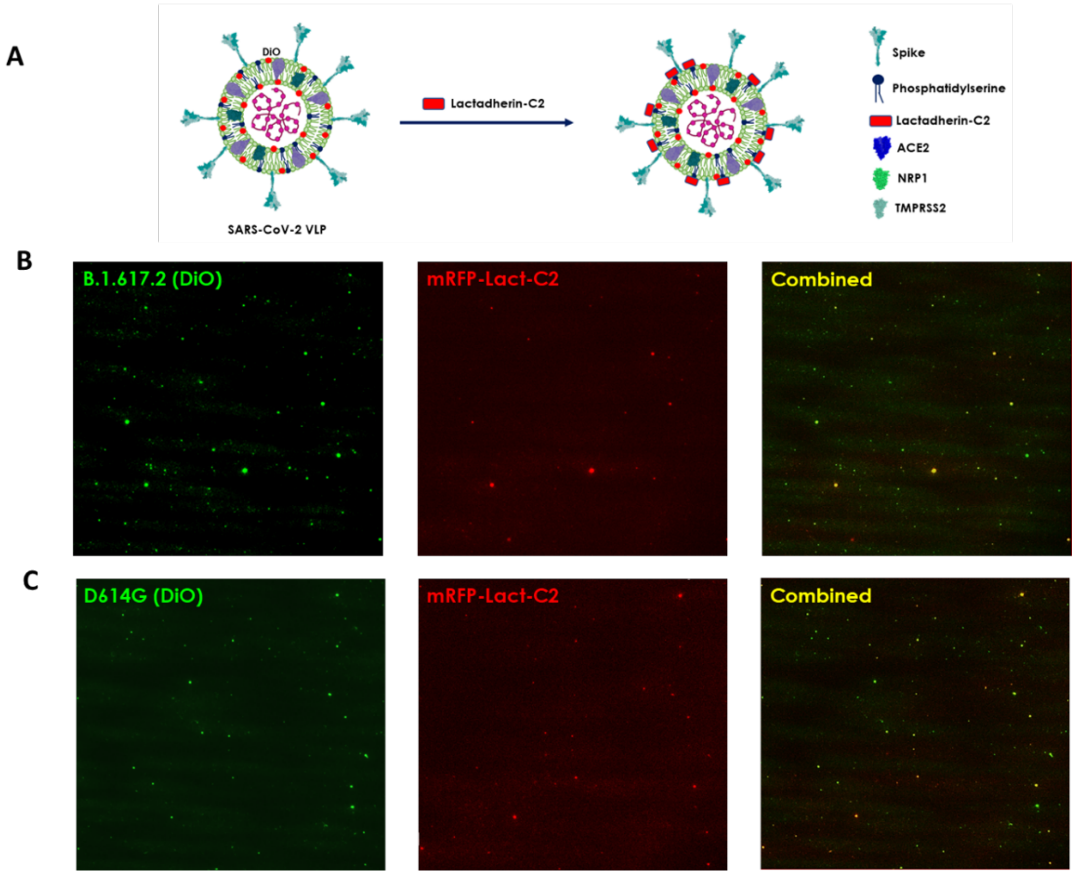

Figure S15. Phosphatidylserine (PS) is present in the SARS CoV-2 SEMN virus like particle (VLP) membrane. (A). Assay figure for colocalization of phosphatidylserine (PS) and lactadherin-C2 (Lact-C2) in SARS CoV-2 VLPs. Virus-like particles (VLPs) with membrane protein, envelope protein and nucleocapsid having the spike of the indicated strain were formed. Virions are tagged with DiO (green) and then incubated with Lact-C2 is tagged with mRFP (red). Colocalization data for the **(B)** B.1.617.2 VLPs and the **(C)** D614G VLPs indicates that PS is present on the surface of viral membrane.

Figure S16

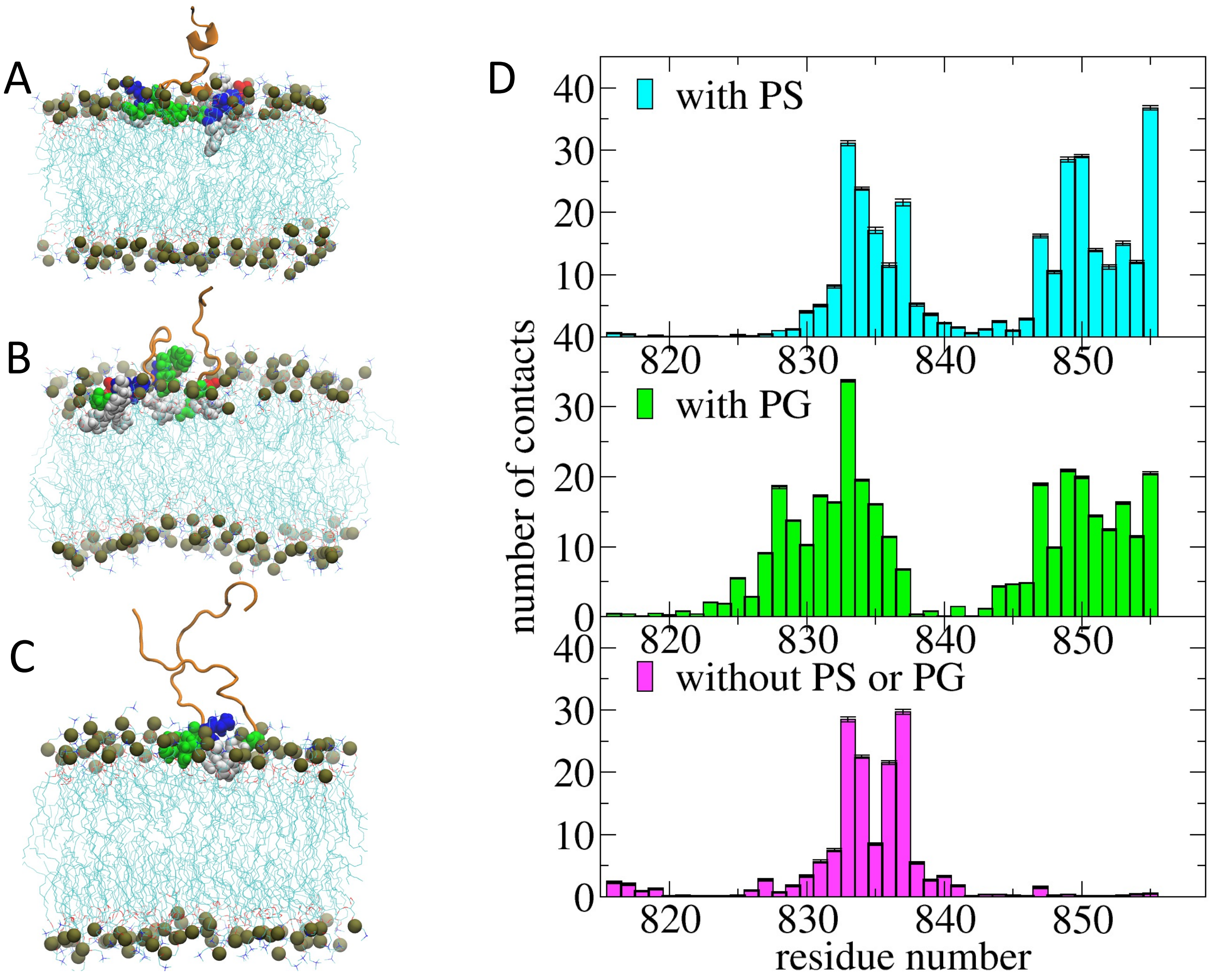

Figure S16. MD simulations of spike FPPR within the lipid bilayers. Equilibrated structure of the S2 fusion peptide proximal residue (816-855 aa) adsorbed on the **(A)** DOPC/POPC/PS/CHOL bilayer, **(B)** DOPC/POPC/PG/CHOL bilayer and **(C)** DOPC/POPC/CHOL bilayer membrane, obtained after 1000 ns long MD simulation. Peptide is shown in the cartoon (brown color) representation. Binding mode of the fusion peptide with PS or PG lipid seems similar but binding of the fusion peptide is significantly less in the absence of PS lipids in the membrane. **(D)** Number of lipid contacts with various residues of the fusion peptide in the presence of PS (top), in presence of PG (middle) and absence (bottom) of PS or PG lipids.

Figure S17

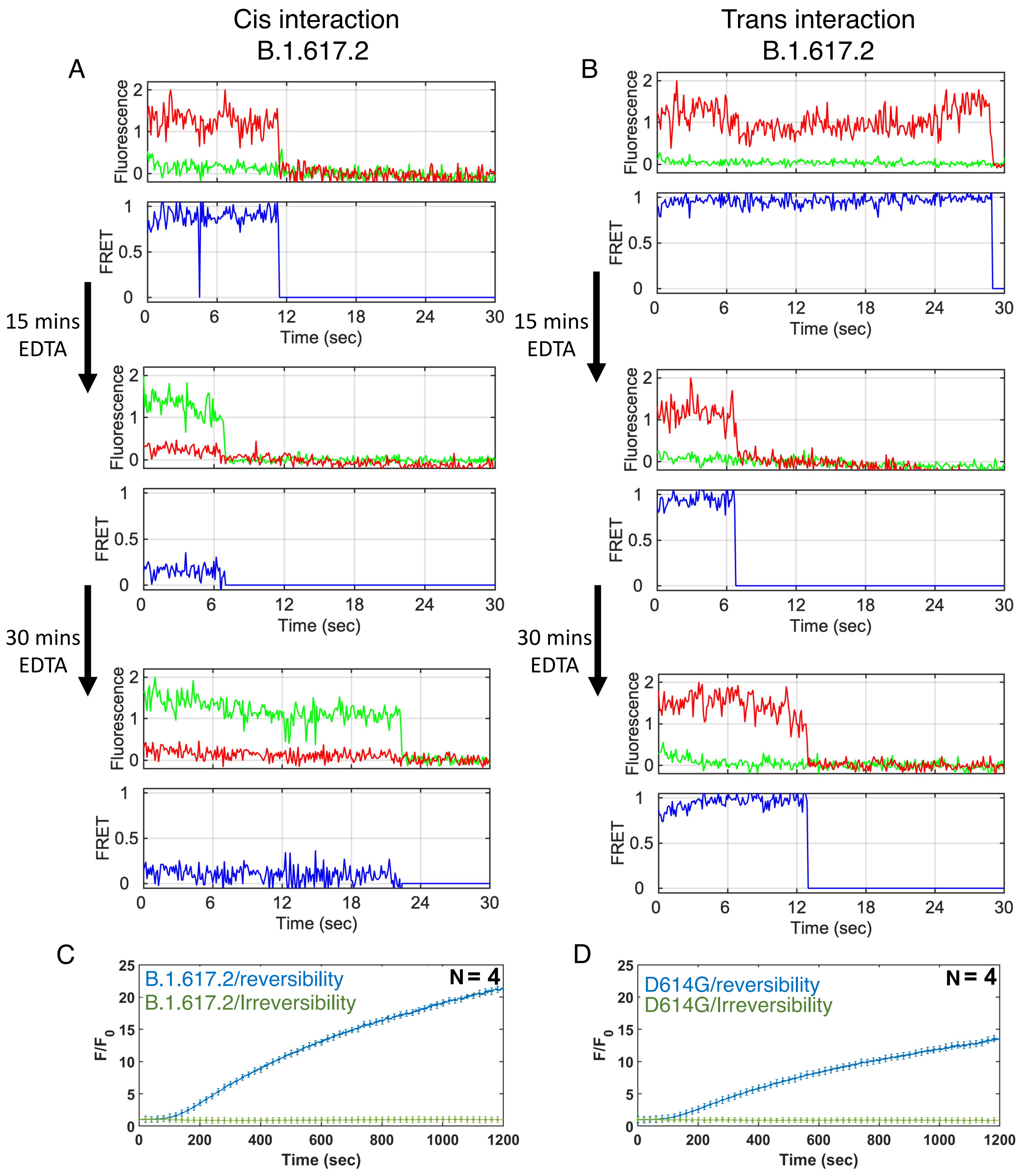

Figure S17. Representative smFRET traces for B.1.617.2 spike showing the FRET state reversibility during cis interaction and FRET state irreversibility during trans interaction. (A) Representative fluorescence (donor, green; acceptor, red) and FRET trajectories (blue) obtained from a single B.1.617.2 spike during Cis

interaction for the B.1.617.2 spike pseudovirions and PS lipid. Low pH and Ca^{2+} triggers the B.1.617.2 spike and PS interaction as indicated from the high FRET state. Addition of EDTA after 15 minutes, converts the high FRET state to low FRET state, indicating that the high FRET state obtained in Cis interaction is a reversible state and not a post fusion state. Addition of EDTA after 30 minutes, shows the conversion of high FRET state to low FRET state. **(B)** Representative fluorescence (donor, green; acceptor, red) and FRET trajectories (blue) obtained from a single B.1.617.2 spike during Trans interaction for the B.1.617.2 spike pseudovirions and PS lipid. Low pH and Ca^{2+} triggers the B.1.617.2 spike and PS interaction as indicated from the high FRET state. Addition of EDTA after 15 minutes or after 30 mins doesn't change the FRET state, indicating that the high FRET state obtained in Trans interaction is an irreversible state and a post fusion state. **(C,D)** lipid mixing data shows that the reversible low FRET state is functionally active for lipid mixing whereas spike after trans interaction is not functionally active.
