# Supplementary material for: SARS-CoV-2 spike fusion peptide trans interaction with phosphatidylserine lipid triggers membrane fusion for viral entry
Source: mBio. 2024 Aug 8;15(9):e01077-24. doi: 10.1128/mbio.01077-24 (PMC11389415; doi:10.1128/mbio.01077-24)
Supplement: Table S1 — Fitting parameters of D614G, B.1.1.7, B.1.351, and B.1.617.2 fusion with proteoliposome in presence of PS lipid. [file mbio.01077-24-s0002.docx]

**Table S1: Fitting parameters of D614G, B.1.1.7, B.1.351 and B.1.617.2 fusion with proteoliposome in presence of PS lipid. The dequenching data related to fusion were fitted to the exponential function A (1- exp-(ktn)).**

| **System** | **A** | **k** | **n** | **R-squared** |
| --- | --- | --- | --- | --- |
| **B.1.617.2/lipo-PS/ACE2-NRP1-TMPRSS2/pH 4.6/Ca** | 90.25±0.76 | 0.000372  ±0.0000546 | 1.307±0.026 | 0.996 |
| **B.1.1.7/lipo-PS/ACE2-NRP1-TMPRSS2/pH 4.6/Ca** | 57.58±0.85 | 0.03353±0.06712 | 0.6816  ±0.0005287 | 0.987 |
| **B.1.351/lipo-PS/ACE2-NRP1-TMPRSS2/pH 4.6/Ca** | 51.38±0.68 | 0.01883±0.03767 | 0.833±1.6656 | 0.9963 |
| **D614G /lipo-PS/ACE2-NRP1-TMPRSS2/pH 4.6/Ca** | 40.71±0.15 | 4.05E-05±0.00002 | 1.544±0.014 | 0.9969 |
