# Supplementary material for: SARS-CoV-2 spike fusion peptide trans interaction with phosphatidylserine lipid triggers membrane fusion for viral entry
Source: mBio. 2024 Aug 8;15(9):e01077-24. doi: 10.1128/mbio.01077-24 (PMC11389415; doi:10.1128/mbio.01077-24)
Supplement: Table S2 — Fitting parameters of D614G, B.1.1.7, B.1.351, and B.1.617.2 fusion with proteoliposome in presence of varying concentrations of Lact-C2. [file mbio.01077-24-s0003.docx]

**Table S2: Fitting parameters of D614G, B.1.1.7, B.1.351 and B.1.617.2 fusion with proteoliposome in presence of varying concentration of Lact-C2. The dequenching data related to fusion were fitted to the exponential function A (1- exp-(ktn)).**

| **System** | **A** | **k** | **n** | **R-squared** |
| --- | --- | --- | --- | --- |
| **D614G /lipo-PS/ACE2-NRP1-TMPRSS2/pH 4.6/Ca/0 pM Lact-C2** | 40.71±0.15 | 4.05E-05±0.00002 | 1.544±0.014 | 0.9969 |
| **D614G /lipo-PS/ACE2-NRP1-TMPRSS2/pH 4.6/Ca/10 pM Lact-C2** | 33.51±0.47 | 1.118±0.0056 | 0.3678±0.73 | 0.9236 |
| **D614G /lipo-PS/ACE2-NRP1-TMPRSS2/pH 4.6/Ca/50 pM Lact-C2** | 2.446±0.559 | 0.008546±0.004205 | 0.7336±0.1313 | 0.9319 |
|  | | | | |
| **B.1.351/lipo-PS/ACE2-NRP1-TMPRSS2/pH 4.6/Ca/0 pM Lact-C2** | 51.38±0.68 | 0.01883±0.03767 | 0.833±1.6656 | 0.9963 |
| **B.1.351/lipo-PS/ACE2-NRP1-TMPRSS2/pH 4.6/Ca/10 pM Lact-C2** | 25.94±0.36 | 0.0289±0.00578 | 0.6941±0.005848 | 0.9887 |
| **B.1.351/lipo-PS/ACE2-NRP1-TMPRSS2/pH 4.6/Ca/50 pM Lact-C2** | 11.732±0.52 | 0.03401±0.0444 | 0.6345±0.02916 | 0.9826 |
| **B.1.351/lipo-PS/ACE2-NRP1-TMPRSS2/pH 4.6/Ca/100 pM Lact-C2** | 11.58±1.59 | 4.522e-07±0.0675e-07 | 2.161±0.224 | 0.9386 |
|  | | | | |
| **B.1.1.7/lipo-PS/ACE2-NRP1-TMPRSS2/pH 4.6/Ca/0 pM Lact-C2** | 57.58±0.85 | 0.03353±0.06712 | 0.6816  ±0.0005287 | 0.987 |
| **B.1.1.7/lipo-PS/ACE2-NRP1-TMPRSS2/pH 4.6/Ca/10 pM Lact-C2** | 31.32±0.467 | 0.03099±0.00006199 | 0.7727±0.00467 | 0.895 |
| **B.1.1.7/lipo-PS/ACE2-NRP1-TMPRSS2/pH 4.6/Ca/50 pM Lact-C2** | 18.02±0.0065 | 0.003055±0.0000024 | 0.4847±0.0078 | 0.952 |
| **B.1.1.7/lipo-PS/ACE2-NRP1-TMPRSS2/pH 4.6/Ca/100 pM Lact-C2** | 15.678±0.012 | 0.02513±0.00047 | 0.6735±0.02269 | 0.9757 |
| **B.1.1.7/lipo-PS/ACE2-NRP1-TMPRSS2/pH 4.6/Ca/200 pM Lact-C2** | 6.192±0.07 | 0.001159±0.00012 | 1.096±0.002 | 0.9972 |
|  | | | | |
| **B.1.617.2/lipo-PS/ACE2-NRP1-TMPRSS2/pH 4.6/Ca/0 pM Lact-C2** | 90.25±0.76 | 0.000372  ±0.0000546 | 1.307±0.026 | 0.996 |
| **B.1.1.7/lipo-PS/ACE2-NRP1-TMPRSS2/pH 4.6/Ca/10 pM Lact-C2** | 30.29±0.968 | 6.111e-08±1.658e-09 | 2.471±0.18 | 0.937 |
| **B.1.1.7/lipo-PS/ACE2-NRP1-TMPRSS2/pH 4.6/Ca/50 pM Lact-C2** | 21.768±0.659 | 0.01155±0.0007091 | 0.8991±0.126 | 0.9634 |
| **B.1.1.7/lipo-PS/ACE2-NRP1-TMPRSS2/pH 4.6/Ca/100 pM Lact-C2** | 21.49±0.0086 | 7.339e-05±4.687e-06 | 0.5115±0.218 | 0.8993 |
| **B.1.1.7/lipo-PS/ACE2-NRP1-TMPRSS2/pH 4.6/Ca/200 pM Lact-C2** | 11.708±0.000227 | 0.6761±0.003372 | 0.8593±0.00007778 | 0.9398 |
| **B.1.1.7/lipo-PS/ACE2-NRP1-TMPRSS2/pH 4.6/Ca/500 pM Lact-C2** | 10.938±0.0638 | 0.0007499±2.082e-04 | 0.4543±0.005833 | 0.9279 |
| **B.1.1.7/lipo-PS/ACE2-NRP1-TMPRSS2/pH 4.6/Ca/1 nM Lact-C2** | 10.029±0.00739 | 1.523e-05±1.946e-07 | 0.7321±0.008 | 0.9307 |
| **B.1.1.7/lipo-PS/ACE2-NRP1-TMPRSS2/pH 4.6/Ca/5 nM Lact-C2** | 3.135±0.00665 | 0.00435±0.0000097 | 1.689±0.0058 | 0.9257 |
